# Quantitative proteomics analysis of the Arg/N-end rule pathway of targeted degradation in Arabidopsis roots

**DOI:** 10.1002/pmic.201400530

**Published:** 2015-04-17

**Authors:** Hongtao Zhang, Lucy Gannon, Stephen J Powers, Kathryn S Lilley, Frederica L Theodoulou

**Affiliations:** 1 Biological Chemistry and Crop Protection Department, Rothamsted ResearchHarpenden, UK; 2 Cambridge Centre for Proteomics, Cambridge Systems Biology Centre, University of CambridgeCambridge, UK; 3 Computational and Systems Biology Department, Rothamsted ResearchHarpenden, UK

**Keywords:** N-end rule, Plant proteomics, Quantitative proteomics, Root, TMT, TAILS

## Abstract

According to the Arg/N-end rule pathway, proteins with basic N-termini are targeted for degradation by the *Arabidopsis thaliana* E3 ligase, PROTEOLYSIS6 (PRT6). Proteins can also become PRT6 substrates following post-translational arginylation by arginyltransferases ATE1 and 2. Here, we undertook a quantitative proteomics study of Arg/N-end rule mutants, *ate1/2* and *prt6*, to investigate the impact of this pathway on the root proteome. Tandem mass tag labelling identified a small number of proteins with increased abundance in the mutants, some of which represent downstream targets of transcription factors known to be N-end rule substrates. Isolation of N-terminal peptides using terminal amine isotope labelling of samples (TAILS) combined with triple dimethyl labelling identified 1465 unique N-termini. Stabilising residues were over-represented among the free neo-N-termini, but destabilising residues were not markedly enriched in N-end rule mutants. The majority of free neo-N-termini were revealed following cleavage of organellar targeting signals, thus compartmentation may account in part for the presence of destabilising residues in the wild-type N-terminome. Our data suggest that PRT6 does not have a marked impact on the global proteome of Arabidopsis roots and is likely involved in the controlled degradation of relatively few regulatory proteins. All MS data have been deposited in the ProteomeXchange with identifier PXD001719 (http://proteomecentral.proteomexchange.org/dataset/PXD001719).

## 1 Introduction

In eukaryotic cells, controlled degradation plays a pivotal role in determining protein abundance throughout signalling and development [1]. During regulated proteolysis, proteins are covalently modified by addition of chains of ubiquitin molecules to internal lysine residues, tagging them for degradation by the 26S proteasome. This reaction is catalysed by E3 ligases, which also determine selectivity by recognising a degradation signal (known as the degron) in a protein substrate. The amino-terminal (Nt) residue of a protein was one of the first classes of degrons to be identified, leading to the discovery of the “N-end rule,” which states that the half-life of a protein is determined by its Nt amino acid [2]. There are two known branches of the N-end rule pathway: the recently discovered Ac/N-end rule, which targets acetylated (Ac) Nt residues and the Arg/N-end rule that recognises unacetylated N-termini (Supporting Information Fig. 1) [3–6]. Between 70 and 90% of eukaryotic proteins are N-terminally (*N-α*)-acetylated and classically, N-terminal acetylation has been associated with protein stability [7,8]. However, Varshavsky and collaborators have shown that acetylated Met is targeted for degradation when followed by a bulky hydrophobic residue (Ac-MΦ) and residues that are acetylated following Nt methionine excision (NME) also behave as Ac/N-degrons if they are not shielded by correct protein folding and/or assembly into oligomeric complexes (Supporting Information Fig. 1). In this way, the Ac/N-end rule plays important functions in the regulation of subunit stoichiometry and general protein quality control [9,10].

**Figure 1:**
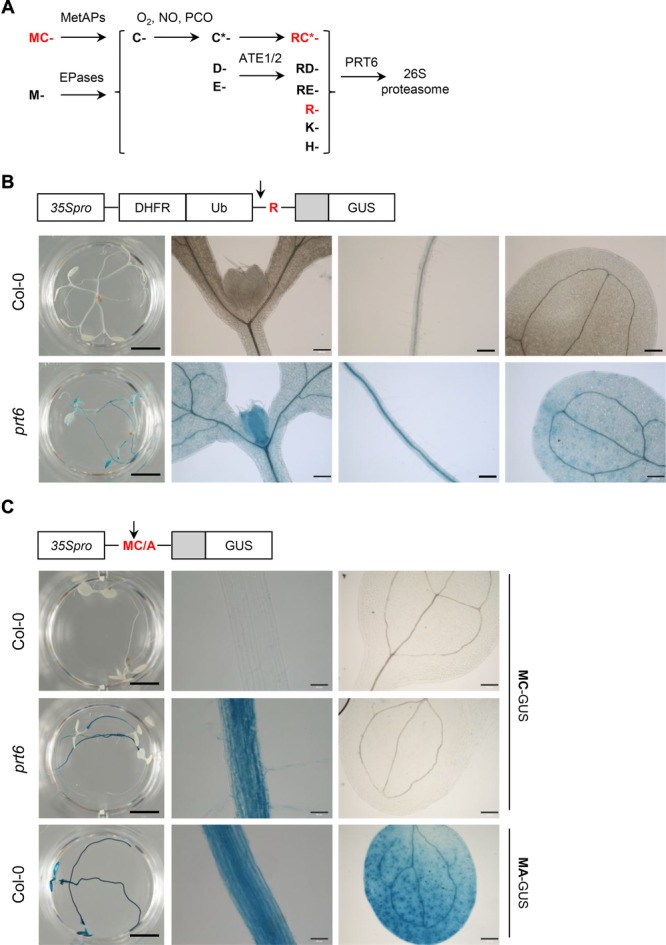
The Arg/N-end rule is active in Arabidopsis roots. (A) Schematic representation of the PRT6 branch of the Arg/N-end rule in Arabidopsis. Proteins enter the pathway following cleavage by endopeptidases (EPases) or methionine aminopeptidases (MetAPs) in the case of Met-Cys proteins. C* indicates oxidized Cys, catalyzed by plant cysteine oxidases (PCO). (B) Expression of R-GUS protein stability reporter in Col-0 and *prt6* seedlings. In planta, the fusion protein is cleaved by ubiquitin-specific proteases (indicated by arrow) to remove dihydrofolate reductase-ubiquitin (DHFR-Ub), generating a variant of GUS that is preceded by an unstructured region (light gray) with an Nt R residue. From left to right: whole seedling (scale bar: 0.5 cm); leaf/cotyledon axil, root, cotyledon (scale bars: 200 μm). (C) Expression of MC-GUS in 5 day old seedlings of *prt6* and Col-0 and MA-GUS in Col-0. Met1 is removed cotranslationally by MetAPs, as indicated by the arrow. The MC-GUS/*prt6* line was back-crossed to Col-0 to enable a direct comparison of the same transgene event in wild-type and mutant backgrounds. From left to right: whole seedling (scale bar: 0.5 cm); root (scale bar: 50 μm); cotyledon (scale bar: 200 μm). Cartoons show schematics of DNA constructs.

In the Arg/N-end rule, the Nt residue is classified as stabilising or destabilising, depending on the fate of the protein. Proteins are synthesised with Nt Met, therefore N-degrons containing destabilising residues are created through proteolytic cleavage following translation and can also be generated via successive enzymatic modifications to the N-terminus of a protein. Thus, the N-end rule is hierarchically arranged (Fig. 1A; Supporting Information Fig. 1) [4]. In eukaryotes, bulky, hydrophobic and basic residues constitute primary destabilising residues that are recognised by distinct classes of E3 ligases, known as N-recognins. Acidic amino acids (Asp and Glu) are secondary destabilising residues, which can be post-translationally modified by arginyl transferase (ATE) enzymes. Amino acids with amine R-groups (Asn and Gln) are tertiary destabilising residues, being subject to deamidation by specific N-terminal amidases followed by ATE-catalysed arginylation. In mammals and plants, Cys is also a destabilising residue, because nitric oxide (NO)-dependent oxidation renders it susceptible to arginylation. Cys residues may be revealed following endopeptidase cleavage or by the action of methionine aminopeptidases (MetAPs), therefore proteins initiating with methionine-cysteine (MC-proteins) are potential N-end rule substrates [11–14].

The architecture of the Arg/N-end rule pathway is largely conserved between yeast, mammals and plants [[4–6, 12]], but the number and specificity of enzymatic components varies. The prototypic N-recognin is *Saccharomyces cerevisiae* Ubr1 [4]. Ubr1 has three substrate-recognition domains: types 1 and 2 domains that recognise N-degrons with basic and hydrophobic residues, respectively and a third domain that recognises an internal degron in CUP9 [4]. In yeast, Ubr1 also promotes degradation of misfolded cytosolic proteins [10, and references therein]. In contrast, *Arabidopsis thaliana* has at least three N-recognins with distinct substrate specificities: PROTEOLYSIS1 and PRT6 that recognise aromatic and basic N-termini, respectively, and an as yet unidentified E3 ligase that handles degrons with hydrophobic N-termini [15]. PRT6 shares several conserved domains with Ubr1, including the UBR box that binds basic N-termini and a RING finger domain, but lacks the type 2 recognition domain [15].

Genetic and biochemical studies have demonstrated that turnover of regulatory proteins by the Arg/N-end rule pathway plays a range of diverse and important roles in different organisms (reviewed in [4–6]). For example, a functional Arg/N-end rule is required for chromosome separation, neurogenesis, cardiac development, spermatogenesis, apoptosis, DNA repair and the sensing of small molecules such as haem [4,5]. In plants, the PRT6 branch of the pathway functions in the control of germination, seed oil mobilisation, leaf development and hormone signalling [16,17] and ATE1 has been implicated in leaf senescence [18]. Intriguingly, N-end rule protein substrates initiating with MC- act as sensors of oxygen and NO by virtue of enzyme-catalysed Cys2 oxidation. Although the sensing mechanism appears to be conserved, the sensor proteins are organism-specific: type VII ethylene response factor (ERF) transcription factors in plants and regulators of G-protein signalling in mammals [11,13,14,19–21].

Despite the importance of the Arg/N-end rule pathway, only a handful of protein substrates have been identified, artificial substrates having largely been used to define the architecture of the pathway [4,12,15]. In this study, we analysed the impact of the PRT6-dependent branch of the Arg/N-end rule on the proteome of *A. thaliana*. The availability of *prt6* null alleles and a double mutant (*ate1-2 ate2-1*) that lacks arginyl t-RNA transferase activity enabled a quantitative proteomics approach to identify proteins that are regulated by the Arg/N-end rule. Using tandem mass tag (TMT) labelling, we identified proteins with altered abundance in N-end rule mutants and found that relatively few proteins were markedly affected. While instructive, this global approach does not provide information about protein cleavage events that potentially create N-degrons. In recent years however, several powerful positional proteomics techniques have been developed for the identification of protein N-termini, including negative selection approaches such as combined fractional diagonal chromatography (COFRADIC) [22,23] and terminal amine isotopic labelling of substrates (TAILS) [24–26]. Here, we combined TAILS with triple dimethyl labelling for identification and quantification of protein N-termini in Arabidopsis. This enabled a detailed analysis of the root N-terminal proteome, revealing extensive posttranslational protein cleavage events in plants.

## 2 Materials and methods

### 2.1 N-end rule reporter plants and mutant alleles

*prt6-1, prt6-5* and the *ate1-2 ate2-1* double mutant (hereafter referred to as *ate1/2*) are described in [16,17]. The construction of GUS initiating with arginine (R-GUS) and MC-GUS plants is described in [14,15].

### 2.2 Plant growth and seedling treatments

Seeds were surface-sterilised and plated on 0.5 × MS medium containing 0.5% sucrose. After 2 days dark chilling (4°C), plates were transferred to a controlled environment cabinet for 5 days (18 h day; 6 h night, 22°C).

### 2.3 Tandem mass Tag™ (TMT) labelling and protein quantification

Methods for root proteome preparation, TMT labelling, mass spectrometry and statistical treatment of data are given in Supplementary data.

### 2.4 Dimethyl labelling and enrichment of N-termini by TAILS

Protein N-termini were enriched from 5 day old roots using a protocol based on [25] and [27]. Protein was extracted in buffer containing 6M Guanidine hydrochloride (GuHCl), 100 mM HEPES (pH 7.5), Complete Mini protease inhibitor Cocktail (Roche), PhosSTOP Phosphatase Inhibitor Cocktail (Roche) and 100 μM MG-132. Protein aliquots (2 mg) were reduced with 10 mM DTT for 1 h at 50°C and alkylated with 15 mM iodoacetamide for 30 min in the dark at RT. Iodoacetamide was quenched with 5 mM DTT for 10 min at RT. Protein aliquots (600 μg) were subjected to dimethyl labelling in 100 mM HEPES to pH 7.0 with the following reagents (Sigma): 40 mM formaldehyde (CH_2_O), 20 mM NaBH_3_CN (“light”); 40 mM deuterated formaldehyde (CD_2_O), 20 mM NaBH_3_CN (“intermediate”); 40 mM heavy formaldehyde (^13^CD_2_O) and 20 mM NaBD_3_CN (“heavy”). Reactions were incubated at 37°C overnight. Glycine was added from a stock solution (1.1 M 100mM HEPES, pH 7.0) to give a final concentration of 100 mM and the reactions incubated at 37°C for 2 h. Three samples were prepared from a single experiment, to permit different labelling combinations: (A) Col-0/light, *prt6*/intermediate and *ate1/2/*heavy; (B) Col-0/heavy, *prt6*/light and *ate1/2/*intermediate; (C) Col-0/intermediate, *prt6*/heavy and *ate1/2/*light. Samples were diluted with water to give a final concentration of 1.5 M GuHCl and precipitated with chloroform and methanol. Pellets were dissolved in 160 μL 6M GuHCl in 50 mM HEPES pH 7.5, diluted to final concentration of 1M GuHCl and the pH was adjusted to 7.5. MS-grade trypsin (Sigma) was added at a ratio of 1:100 protease/protein and incubated at 37°C for 4 h. Then trypsin was added to give a final ratio of 1:50 protease/protein and incubated at 37°C overnight. Digested samples were centrifuged at 14 000 *g* for 5 min at RT. Aliquots were retained for pre-TAILS analysis [27]. Tryptic peptides (500 μg) were mixed with 2 mg hyperbranched polyglycerol aldehyde polymer (http://flintbox.com/public/project/1948/) prepared according to the manufacturer's protocol and coupled by incubation with 20 mM NaBH_3_CN overnight at 37°C. A 100 mM glycine was added the reaction mixture incubated at RT for 2 h then filtered through 10-kDa Microcon spin-filter and desalted using Sep-Pack light C18 (Waters).

### 2.5 LC-MS/MS

LC-MS/MS was performed with a nanoAquity UPLC and an LTQ Orbitrap Velos hybrid ion trap mass spectrometer (Thermo Scientific, Waltham, MA). Details are given in Supplementary Information.

### 2.6 TAILS MS data analysis

Raw data were searched against TAIR10 database using Mascot 2.4 (Matrix Science) and Proteome Discoverer™ version 1.4.1.14 (DBVersion:79; Thermo Scientific), employing Top 10 peaks filter node and percolator nodes. As filters, high peptide confidence corresponding to *q*-values below 0.01 (FDR < 1%), search engine rank 1 and Mascot ion score 20 were used. Dimethyl TAILS experiments employed the precursor ions quantifier using a three-step strategy and semi-ArgC enzyme specificity with a maximum of one missed cleavage with carbamidomethylation of Cys as a fixed modification and dimethylation light (+28.031 Da), dimethylation intermediate (+32.056 Da) and heavy (+36.076Da) at Lys, and oxidized methionine as variable modifications. First, N-terminal acetylation (+42.0105 Da) was set as a variable modification; second, dimethylation light, intermediate and heavy at N-termini were set as variable modifications. Third, a variable modification search was used for dimethyl-arginylation (see Supplementary Information). Mass tolerances were set to 20 ppm for MS and 0.6 Da for MS/MS. For quantification, the maximum window for corresponding peptides was set to 1 min, single-Peak/Missing Channels allowed was set as two. The ratios were normalized by the medians of pre-TAILS samples searched with ArgC enzyme specificity. Peptides were defined as Nt if they were Nt-acetylated or bore an Nt-dimethyl label (corresponding to free Nt peptides). Application of the semi-ArgC search to pre-TAILS data (Supporting Information Table 2) yielded some false-positives since internal peptides bearing an N-terminal Lys residue labelled on the free ε-amino group may be misidentified as Nt-dimethylated with this search. These peptides are retained by the HPG matrix and do not appear in the TAILS dataset, as is evident from the relatively low overlap between TAILS and pre-TAILS free Nt peptides (Supporting Information Fig. 2). Therefore caution is required when interpreting searches of pre-TAILS data. To generate the final list of N-terminal peptides from the TAILS dataset (Supporting Information Table 3), only peptides with N-terminal acetylation or dimethylation modifications and available position information in the protein without conflicting lysine modifications were included. Some peptides do not end with Arg, contrary to the ArgC specificity of trypsin: this could reflect cleavage by endogenous proteases or alternatively might result from hydrolysis in the acidic solution prior to LC-MS. The mass spectrometry proteomics data have been deposited to the ProteomeXchange Consortium [28] via the PRIDE partner repository with the dataset identifier PXD001719.

## 3 Results and discussion

### 3.1 The PRT6 pathway is active in roots

The expression and activity of PRT6 were investigated in order to select a suitable tissue for proteomics analysis. Public microarray data indicated that *PRT6*, *ATE1* and *ATE2* transcripts were expressed at a low level throughout the plant (Supporting Information Fig. 3). The location of PRT6 activity was tested using two different protein stability reporters: firstly, a reporter bearing a primary destabilising residue, Arg-GUS (R-GUS) was generated by the ubiquitin fusion technique [15]. R-GUS was unstable in wild-type (Col-0) plants, but was detected throughout *prt6* seedlings (Fig. 1B). Transgenic plants expressing MC-GUS under the control of the constitutive Cauliflower mosaic virus 35S promoter were then used to report the cumulative outcome of sequential NME, Cys oxidation, arginylation and PRT6-dependent degradation. Methionine-alanine-GUS served as a stable control. MC-GUS was unstable in Col-0 but stabilised in roots of *prt6* and *ate1/2* seedlings (Fig. 1C and Supporting Information Fig. 4). Taken together, these results indicate that PRT6 is active in roots, cotyledons and leaves but that removal of PRT6 or ATE1/2 was insufficient to stabilise MC-GUS in cotyledons. Therefore roots were selected for further experiments to determine the impact of ATE1/2 and PRT6 on the proteome.

### 3.2 Identification of proteins differentially regulated in N-end rule mutants by TMT labelling

**Figure 2:**
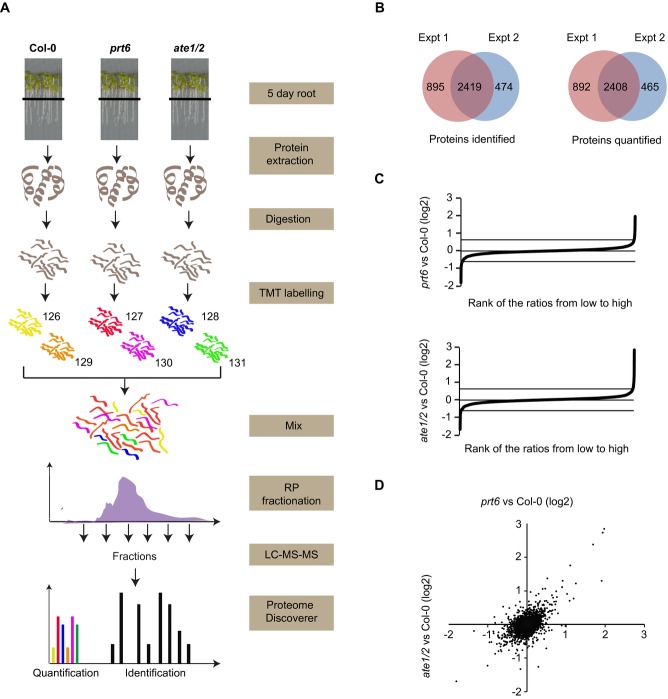
The Arg/N-end rule does not cause major perturbations in the proteome of roots. (A) Schematic representation of the TMT workflow. A label-swap was performed for the second experiment. (B) Venn diagram showing proteins identified and quantified in two independent experiments. (C) Plots of changes in protein abundance in N-end rule mutants, *prt6* and *ate1/2* relative to Col-0. Plots depict log (2) transformed ratios of abundance of 3765 proteins ranked from low to high. (D) Scatter plot of the log (2) transformed ratios (*prt6*: Col-0 versus *ate1/2*: Col-0) for 3765 proteins. See Table 1 and Supporting Information Table 1 for list of proteins.

Labelling of root proteins with TMTsixplex™ reagents was employed to identify proteins with altered abundance in N-end rule mutants (Fig. 2A). The full dataset is presented in Supporting Information Table 1. A total of 3300 proteins were quantified in experiment 1 and 2863 in experiment 2, with an overlap of 2408 (Fig. 2B), however, the vast majority of these proteins did not change in abundance in the mutants (Fig. 2C). Comparison of protein abundance in *prt6* with that in *ate1/2* revealed a relatively close relationship (Fig. 2D), consistent with the fact that these components act sequentially in the degradation pathway. However, not all proteins displayed the same behaviour in both mutants, for example only a subset of proteins up-regulated in *prt6* were also increased in abundance in *ate1/2*, possibly representing proteins that do not require arginylation to be processed by the N-end rule (see Fig. 1A).

Table 1 lists proteins with increased abundance in the mutants. Seventeen up-regulated proteins were identified in both experiments, four of which were previously shown to be up-regulated at the transcriptional level in both *prt6* and *ate1/2* [13]. Some of the genes encoding these proteins are targets of group VII MC-ethylene response factors (MC-ERFs) that are known substrates of PRT6 and ATE1/2 [13]. Indeed, At4g27450, At2g16060, At3g03270 and At5g19550 belong to the so-called “core 49” hypoxia-responsive gene set that is regulated by the plant N-end rule [13]. A larger number of putatively up-regulated proteins were identified in only one of the two TMT experiments (Supporting Information Table 1). Whilst it is not possible to assign a statistical significance to these observations, four of the identified proteins [alcohol dehydrogenase 1 (At1g77120), plant cysteine oxidase 1 (PCO1) (At5g15120), pyruvate decarboxylase (At4g33070) and sucrose synthase 1 (At5g20830)] are also transcriptionally up-regulated in *prt6* and *ate1/2* seedlings and belong to the hypoxia-responsive “core 49” [13] and another (At3g11930) is hypoxia-responsive at the transcript level [29], suggesting that at least some of the single observations are biologically significant.

**Table 1:** Proteins with increased abundance in N-end rule mutants

Accession	Description	Ratio *prt6*: Col-0	Ratio *ate1/2*: Col-0
**AT4G27450.1**	**Aluminium induced protein with YGL and LRDR motifs**	3.826**	6.642***
**AT2G16060.1**	**hemoglobin 1**	3.218*	5.200**
**AT3G03270.1**	**Adenine nucleotide alpha hydrolases-like superfamily protein**	2.143*	3.373**
AT2G38380.1	Peroxidase superfamily protein	1.405	1.633*
AT4G26010.1	Peroxidase superfamily protein	1.381*	1.197
**AT5G19550.1**	**aspartate aminotransferase 2**	1.364**	1.629***
AT5G26280.1	TRAF-like family protein	1.344*	1.490*
AT5G23580.1	calmodulin-like domain protein kinase 9	1.342*	1.189
AT1G52070.1	Mannose-binding lectin superfamily protein	1.314**	1.201*
AT5G63550.2	DEK domain-containing chromatin associated protein	1.279*	1.404**
AT3G12580.1	heat shock protein 70	1.198*	1.319**
AT5G61210.1	soluble N-ethylmaleimide-sensitive factor adaptor protein 33	1.194*	1.331**
AT5G12110.1	Glutathione S-transferase, C-terminal-like; Translation elongation factor EF1B/ribosomal protein S6	1.152*	1.454***
AT4G08770.1	Peroxidase superfamily protein	1.127*	1.349**
AT3G07720.1	Galactose oxidase/kelch repeat superfamily protein	1.126*	1.379**
AT1G27450.3	adenine phosphoribosyl transferase 1	1.094	1.338*
AT2G21580.1	Ribosomal protein S25 family protein	0.920	1.327*

The table shows the normalised ratios of protein abundance, determined by TMT labelling of proteins extracted from 5 day old roots (average of two biological replicates). Increased abundance was defined as a 1.3-fold change for either mutant, relative to Col-0. *p < 0.05; **p < 0.01; ***p < 0.001. The *F*-test cut-off was < 0.1. Accessions indicated in bold are transcriptionally upregulated in *prt6* and *ate1/2* [13]. Proteins with increased abundance in N-end rule mutants that were identified in only one experiment are included in Supporting Information Table 1.

We speculate that proteins that are increased in abundance in mutants without a corresponding change in transcript are candidate PRT6 substrates, since their degradation would be impaired in *prt6*. However, it is not possible to claim categorically that this is the case without supporting information, such as evidence that these proteins undergo cleavage to reveal destabilising N-termini and mutation of the cleavage sites causes stabilisation in vivo. It should also be noted that the proteins identified above that are known to be regulated by the MC-ERF transcription factors may also be N-end rule substrates: following their synthesis under hypoxic conditions, these proteins would need to be removed along with ERFs upon reoxygenation to avoid deleterious effects of a prolonged hypoxia response, although further experimentation would be required to test this hypothesis.

MC-ERFs, the only known substrates of PRT6, were not identified in the TMT data set, which is perhaps not surprising since these transcription factors are expected to be of low abundance. Of the ∼250 MC-proteins encoded by the Arabidopsis genome, ten were identified and quantified in the TMT dataset, but were not markedly up-regulated (Supporting Information Table 1). Interestingly, several proteins exhibited significantly reduced abundance in the *prt6* and/or *ate1/2* mutants (Supporting Information Table 1). In the majority of cases, this appears not to be the result of transcriptional down-regulation and the mechanism by which these proteins are down-regulated is not clear.

### 3.3 Enrichment of N-terminal peptides by TAILS

**Figure 3:**
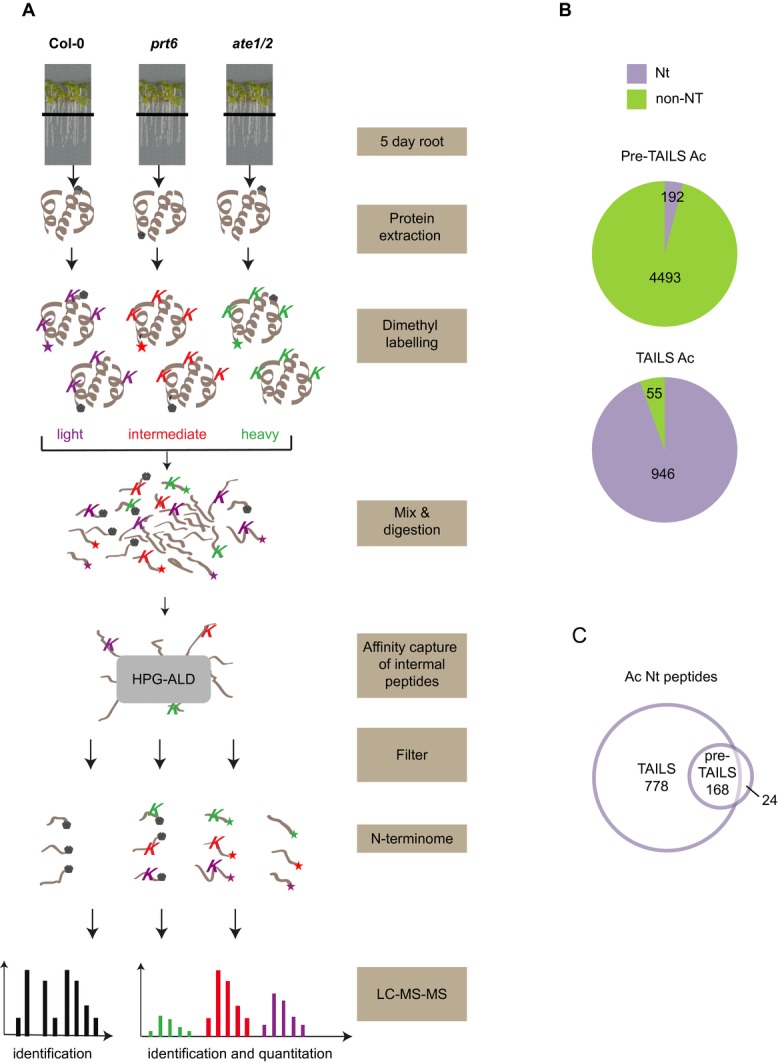
Isolation of N-terminal peptides with dimethyl-TAILS. (A) Schematic representation of the TAILS workflow. Primary amines of proteins with free N-termini (star) and lysine (K) side-chain amines of proteins were chemically modified by isotopically distinct dimethyl labelling (light/intermediate/heavy). After combining labelled samples from WT and N-end rule mutant plants, proteins were digested and internal peptides removed via HPG-ALD polymer binding of the free N-terminal amine group. The unbound peptides (highly enriched for N-terminal peptides) were then analysed and quantified by high-accuracy LC-MS/MS. Mascot and ProteomeDiscoverer™ were used for protein identification and quantification. Grey pentagons represent naturally blocked (acetylated) N-termini. (B) Numbers of unique N-terminal (Nt) acetylated (Ac) and non-Nt peptides identified before (pre-TAILS) and after enrichment by TAILS. (C) Venn diagram showing overlap of Ac Nt peptides identified in pre-TAILS and TAILS samples.

Since the TMT experiments only identified a subset of the proteome, presumably representing abundant proteins, we employed a strategy to reduce sample complexity and to enrich for Nt peptides. Dimethyl TAILS was used to isolate Nt peptides from Col-0, *prt6* and *ate1/2* roots, in order to obtain a snapshot of N-termini present in wild-type samples as well as neo-N-termini generated by the N-end rule (Fig. 3A) [25]. This approach has the advantage that it provides information not only about the identity and abundance of proteins but also about cleavage events that reveal N-degrons and other neo-N-termini [26]. In the starting material (“pre-TAILS” sample; Supporting Information Table 2), searching the dataset with acetylation as a modification identified 4.1% of peptides as N-terminal. Following TAILS enrichment, 94.5% of peptides were identified as Nt acetylated, indicating the efficient depletion of internal peptides during the TAILS procedure (Fig. 3B). The majority of Nt acetylated peptides identified in the pre-TAILS dataset were also present in the TAILS sample (Fig. 3C). Similarly, when we searched for free N-termini using dimethylation as a variable modification, about 96% of peptides were identified as free N-termini in the TAILS sample (Supporting Information Fig. 2A) and the ion score and intensities of TAILS and pre-TAILS samples showed very similar distributions (Supporting Information Fig. 2B, C). Thus TAILS is a highly effective procedure for isolation and identification of Nt peptides.

### 3.4 Acetylated Nt peptides

**Figure 4:**
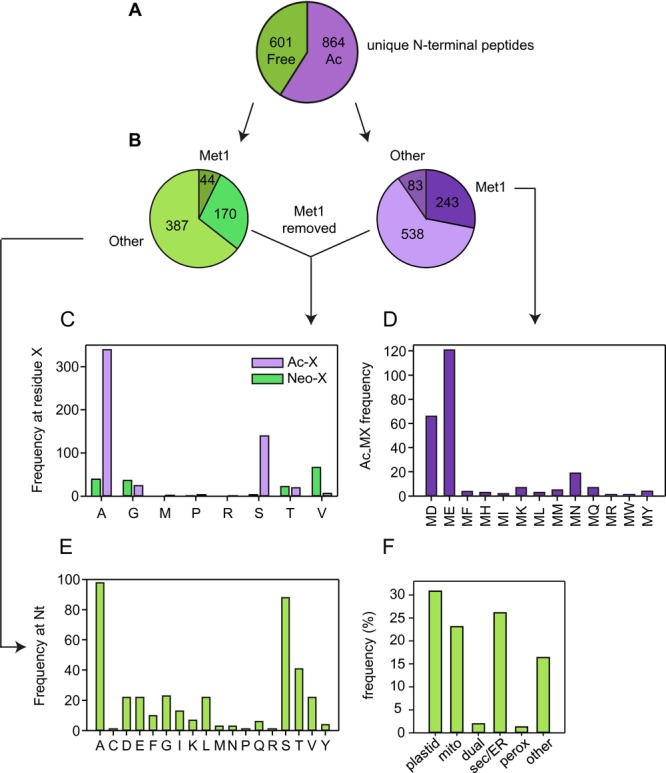
Analysis of unique N-terminal peptides. The dataset is restricted to unique peptides with Nt acetylation or dimethylation and available position information. (A) Pie chart showing the total free and acetylated (Ac) unique N-terminal peptides identified. (B–D) Analysis of first and second residues of neo-N-termini and acetylated N-termini. Nt peptides that initiate at amino acid residue > = 3, relative to the translated protein are designated as “other.” (E) Occurrence of different N-terminal amino acid residues in neo-N-terminal peptides. Only neo-N-terminal peptides where the N-terminus corresponds to residue ≥ 3 of the predicted translated protein were analysed. (F) Percentage of proteins with neo-N-termini in different subcellular locations. Subcellular localisation was assigned based on established annotation or TargetP prediction where annotation was lacking. Mito, mitochondrion; perox, peroxisome; dual, dual-targeted to plastid and mitochondrion; sec/ER, secretory pathway/endoplasmic reticulum.

Of the 1465 unique Nt peptides, 55% were acetylated (Fig. 4A; Supporting Information Table 3). The acetylated N-terminus of ATE1 was present in Col-0 and *prt6* datasets, but absent from *ate1/2*, confirming that *ate1/2* is a null mutant lacking ATE1 protein expression (Supporting Information Table 3). The majority of acetylated Nt peptides were most likely the result of cotranslational Nt acetylation, being acetylated at Met1 or at residue 2 following NME by MetAPs. In yeast, Nt Met has been identified as a primary destabilising residue when it is followed by a bulky hydrophobic residue (MΦ-; Supporting Information Fig. 1) [10]. Ac-MΦ proteins may be degraded through the Ac/N-end rule and non-acetylated MΦ proteins by the Arg/N-end rule. We identified 243 acetylated Nt peptides beginning with Met1 (Fig. 4B). Of these, only 14 (5.76%) were Ac-MΦ peptides (Fig. 4D). Although this is a rather small sample, these peptides appear to be under-represented, compared to their occurrence in Arabidopsis predicted ORFs (ca. 9.52%; Supporting Information Fig. 5) consistent with the possibility that selected Ac-MΦ proteins are degraded in plants as well as yeast.

The bulk of the acetylated Nt peptides identified were generated following NME (Fig. 4B). This dataset conforms to the established specificity of MetAP enzymes and the assertion that A and S at position 2 are most important for NME, with a much lower representation of G,M,P,T and V at position 2 (Fig. 4C) [8]. However, we identified 83 downstream Nt acetylated peptides that initiate at residue 3 or beyond of the original translated protein (defined as “other”). In agreement with the findings of Bienvenut et al. [7], analysis by TargetP [30] revealed that 40% of these represented plastid proteins acetylated following removal of the transit peptide, with a reasonable agreement between TargetP cleavage site prediction and the relative position of acetylated Nt residue (Supporting Information Table 3). Although roots are not photosynthetically competent, they contain specialised plastids with various biosynthetic functions. The remaining downstream Nt acetylated peptides may derive from alternative start sites and other PTMs or cleavage events.

### 3.5 Free Nt peptides

Of 601 free Nt peptides, 44 corresponded to unmodified protein N-termini (Fig.4B). The remainder of the non-acetylated peptides putatively generated by a post-translational cleavage event were classified as “neo” Nt peptides. One hundred and seventy peptides had undergone NME but not Nt acetylation, and 387 initiated at residue 3 or beyond, relative to the predicted translation start (“other”) (Fig.4B, C). W and H, which are predicted to be destabilising [12], were not observed as Nt residues in the latter group, whereas stabilising residues A, S, T and V were highly represented, as would be expected (Fig.4E). A moderate number of Nt peptides with the destabilising residues D, E, F, I, K, L, R and Y was present in the wild-type dataset and these Nt residues were not over-represented in N-end rule mutants, indicating that proteins initiating with destabilising residues are not necessarily targeted for degradation. Indeed, the frequency of different neo-Nt amino acid residues did not differ markedly according to genotype (Supporting Information Fig. 6). Over 80% of the proteins represented in this dataset are predicted to be localised to organelles or the secretory pathway, many neo-Nt peptides evidently having been generated by the removal of targeting sequences (Fig.4F; Supporting Information Table 3). Destabilising residues revealed by signal peptide cleavage would evade 26S proteasomal degradation, by virtue of compartmentation and, although plastids and mitochondria house prokaryotic-like N-end rule pathways, these exhibit a distinct specificity to the classical Arg/N-end rule pathway [31,32].

Interestingly, arginylated peptides were not identified in the *prt6* N-terminome with our search strategy, although the dataset contained numerous neo Nt peptides initiating with Asp or Glu that could potentially be modified in this way (Fig.4E, Supporting Information Fig. 6). This may be because the N-termini are not in the correct conformation or compartment to permit arginylation and it is also possible that arginylation could be subject to feedback regulation in *prt6*. However, the absence of Nt-arginylated proteins from the dataset may simply be a question of coverage. Whilst a study employing immunoaffinity purification revealed widespread post-translational protein arginylation in mouse [33], it is also possible that arginylation is a relatively rare event in plants. In plants as well as in mammals, potential Arg/N-degrons are created by certain classes of metacaspases that cleave adjacent to acidic residues. Isolation by COFRADIC has shown that these N-termini are abundant in plants that have an intact N-end rule, suggesting that arginylation is not an inevitable consequence when Asp or Glu are revealed by protein cleavage [34].

### 3.6 Comparison of TMT and TAILS datasets

We compared the TMT and TAILS data to determine whether any of the proteins with altered abundance in the TMT experiments were represented in the N-terminome. Of the seventeen proteins found by TMT labelling to be significantly up-regulated in N-end rule mutants (Table 1), three were also identified in the TAILS experiment. Acetylated Nt peptides derived from aspartate aminotransferase 2 (At5g19550; Supporting Information Fig. 7), adenine phosphoribosyl transferase 1 (At1g27450) and the protein encoded by At5G12110 were identified by not quantified, owing to lack of an internal lysine residue for dimethyl labelling. The protein encoded by At3g11930 (belonging to the adenine nucleotide alpha hydrolases-like superfamily) was shown to be up-regulated in *prt6* and *ate1/2* both in a single TMT experiment and two replicates of TAILS samples (not detected in other samples; Supporting Information Fig. 7). Of the 26 proteins identified in TMT experiments as significantly down-regulated (1.3-fold) in N-end rule mutants (Supporting Information Table 1), four proteins (At1g62570, At2g01520, At4g23670 and At2G33830) were also found to be down-regulated in the TAILS experiment and (Supporting Information Table 3).

## 4 Concluding remarks

In this study, we explored the role of the Arg/N-end rule on the Arabidopsis root proteome. Current knowledge predicts that protein species with exposed Nt destabilising residues would be efficiently degraded by the 26S proteasome and that a subset of ATE1/2 and PRT6 substrates would be stabilised in N-end rule mutants. However, as recently shown in erythrocytes [27], isolation of Nt peptides revealed that not all proteins bearing destabilising N-termini are efficiently turned over, with compartmentation accounting for the protection of some neo-N-termini. A relatively small number of proteins exhibited markedly increased abundance in *ate1/2* and *prt6* plants, with several of these corresponding to downstream transcriptional targets of known Arabidopsis N-end rule substrates. Furthermore, Nt peptides with destabilising residues were not over-represented in the mutants. Taken together, these data suggest that PRT6 does not have a dramatic impact on the global proteome of Arabidopsis roots. In agreement with this, relatively modest changes in protein abundance were detected in a recent study of N-end rule mutants by parallel reaction monitoring [35].

The only validated Arabidopsis N-end rule substrates to date are Group VII ERF transcription factors [13,14,20], and the function of the PRT6 branch may indeed be to control the abundance of a small number of regulatory proteins. Not all of the phenotypes of *prt6* and *ate1/2* mutants have yet been attributed to stabilisation of ERFs, thus the identification and validation of additional proteins that are degraded via the Arg/N-end rule remains a significant challenge. Future investigations will include fractionation and enrichment for deeper coverage and alternative strategies such as affinity purification of PRT6 and ATE1/2-interacting proteins. However, the approaches presented here represent a useful starting point for the analysis of protein degradation pathways in plants and TAILS has proved to be a highly efficient method for the annotation of the Arabidopsis N-terminome.

## Abbreviations

Ac: acetylated
ATE: arginyl t-RNA transferase
COFRADIC: combined fractional diagonal chromatography
ERF: ethylene response factor
GUS: glucuronidase
MC: methionine-cysteine
NME: N-terminal methionine excision
NO: nitric oxide
Nt: amino-terminal
PRT6: PROTEOLYSIS6
R-GUS: GUS initiating with arginine
TAILS: terminal amine isotopic labelling of substrates
TMT: tandem mass tag

## Supporting Information

As a service to our authors and readers, this journal provides supporting information supplied by the authors. Such materials are peer reviewed and may be re-organized for online delivery, but are not copy-edited or typeset. Technical support issues arising from supporting information (other than missing files) should be addressed to the authors.

Figure S1 Overview of the eukaryotic Arg/N-end rule and the Ac/N-end rule pathways Figure S2. Enrichment and identification of N-terminal peptides by TAILS Figure S3 Abundance of PRT6, ATE1 and ATE2 transcripts in different plant tissues and organs Figure S4 Expression of MC-GUS in ate1 ate2 seedlings Figure S5 Frequency of different amino acid residues at position 2 in Arabidopsis predicted ORFs Figure S6 Analysis of free N-terminal peptides Figure S7 Selected comparisons of TMT and TAILS data Table S1 Proteins identified and quantified using TMT labelling Table S2 Unique peptides identified in pre-TAILS and TAILS samples Table S3 N-terminal peptides identified and quantified using TAILS with position information in the protein and predicted Fsubcellular localization
