# Supplementary material for: Quantitative proteomics analysis of the Arg/N-end rule pathway of targeted degradation in Arabidopsis roots
Source: Proteomics. 2015 Apr 17;15(14):2447–57. doi: 10.1002/pmic.201400530 (PMC4692092; doi:10.1002/pmic.201400530)
Supplement: Supplementary file 1 [file pmic0015-2447-sd1.zip › pmic8103-sup-0001-text.pptx]

## Slide 1
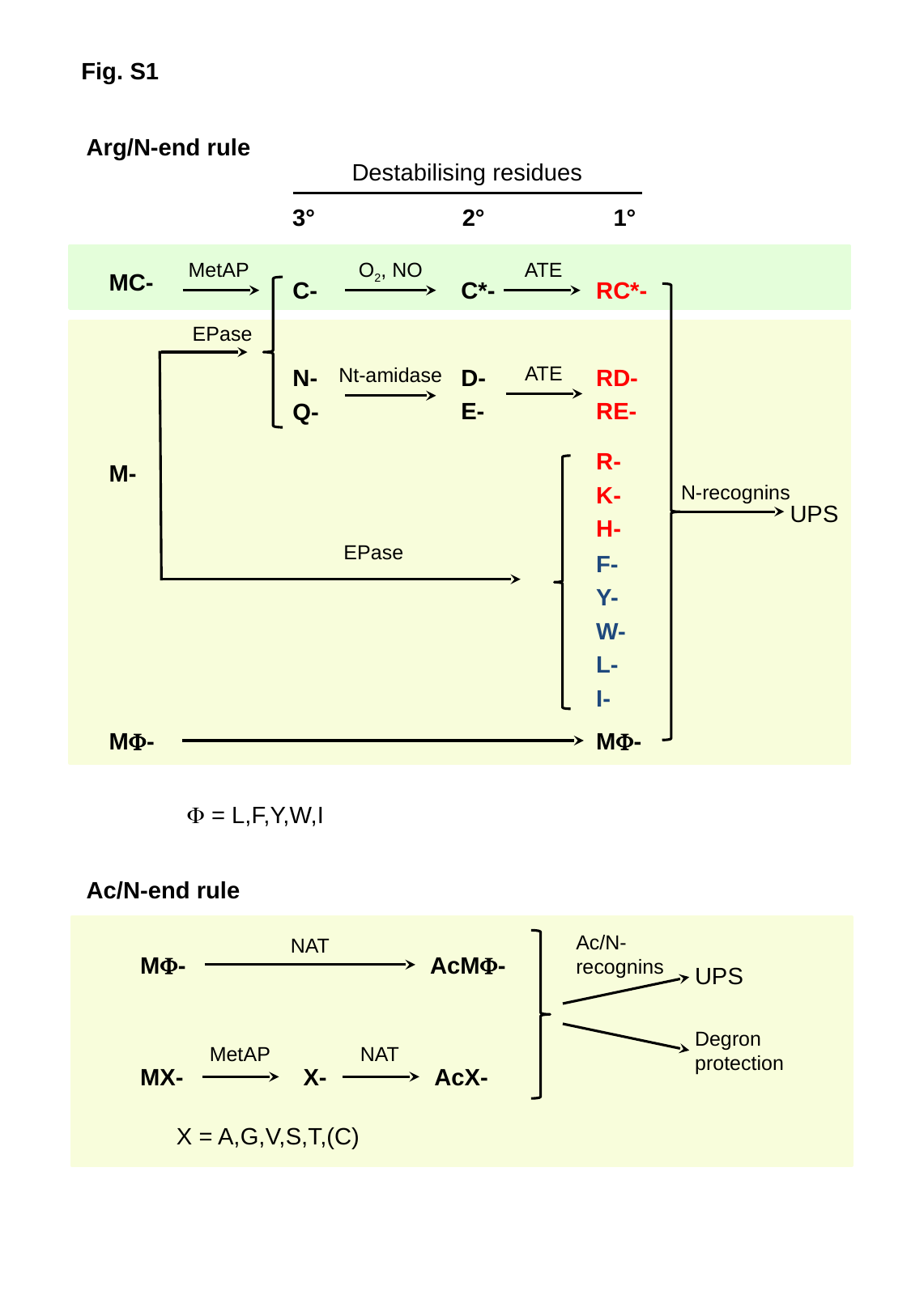

Fig. S1
Arg/N-end rule
Destabilising residues
3°
2°
1°
MetAP
O2, NO
ATE
MC-
C-
C*-
RC*-
EPase
ATE
Nt-amidase
RD-
D-
N-
RE-
E-
Q-
R-
M-
N-recognins
K-
UPS
H-
EPase
F-
Y-
W-
L-
I-
M-
M-
 = L,F,Y,W,I
Ac/N-end rule
Ac/N-recognins
NAT
M-
AcM-
UPS
Degron protection
MetAP
NAT
MX-
X-
AcX-
X = A,G,V,S,T,(C)
