# Supplementary material for: Quantitative proteomics analysis of the Arg/N-end rule pathway of targeted degradation in Arabidopsis roots
Source: Proteomics. 2015 Apr 17;15(14):2447–57. doi: 10.1002/pmic.201400530 (PMC4692092; doi:10.1002/pmic.201400530)
Supplement: Supplementary file 1 [file pmic0015-2447-sd1.zip › pmic8103-sup-0005-text.pptx]

## Slide 1
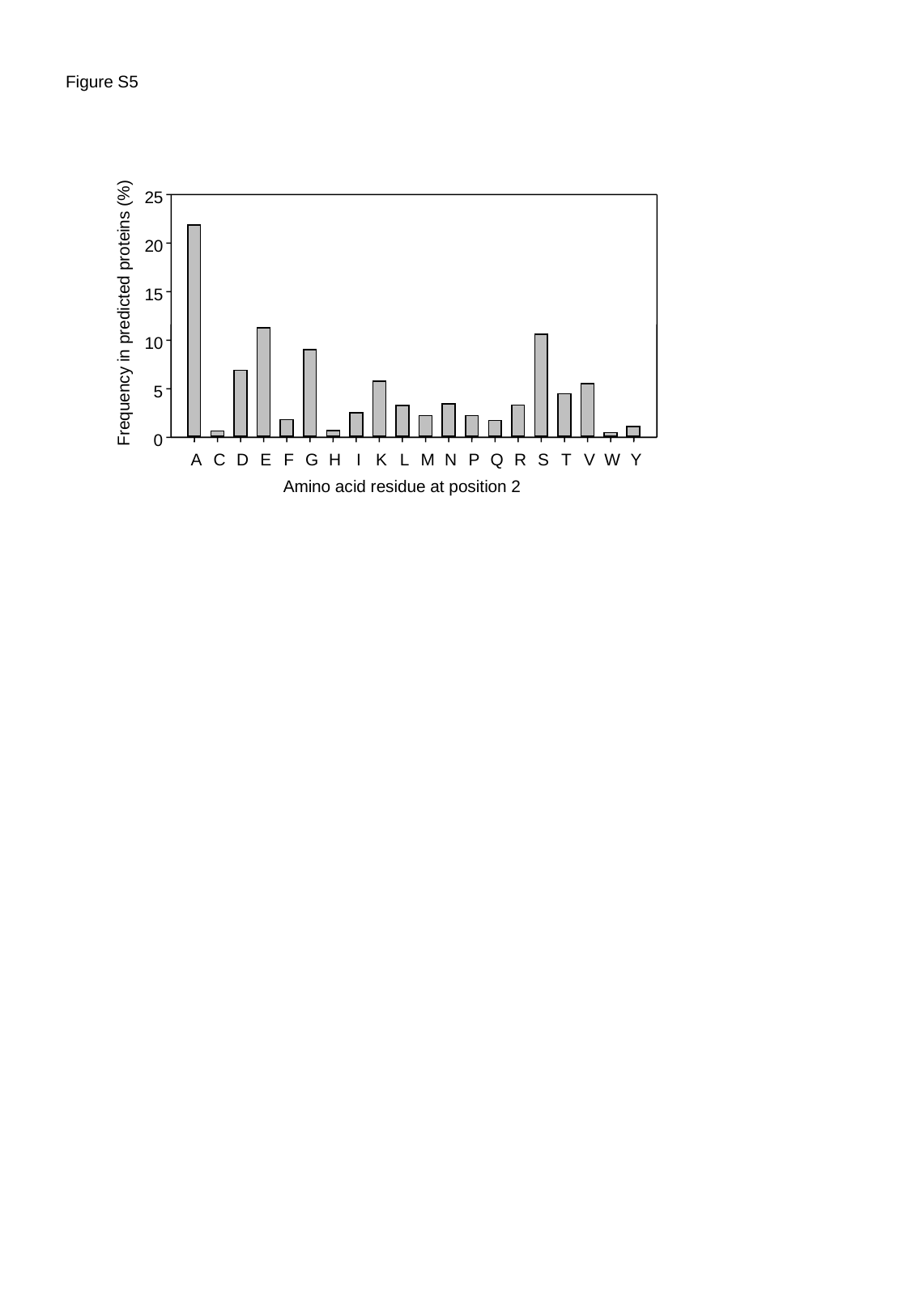

Figure S5
25
20
15
Frequency in predicted proteins (%)
10
5
0
A
C
D
E
F
G
H
I
K
L
M
N
P
Q
R
S
T
V
W
Y
Amino acid residue at position 2
