# Supplementary material for: Quantitative proteomics analysis of the Arg/N-end rule pathway of targeted degradation in Arabidopsis roots
Source: Proteomics. 2015 Apr 17;15(14):2447–57. doi: 10.1002/pmic.201400530 (PMC4692092; doi:10.1002/pmic.201400530)
Supplement: Supplementary file 1 [file pmic0015-2447-sd1.zip › pmic8103-sup-0007-text.pptx]

## Slide 1
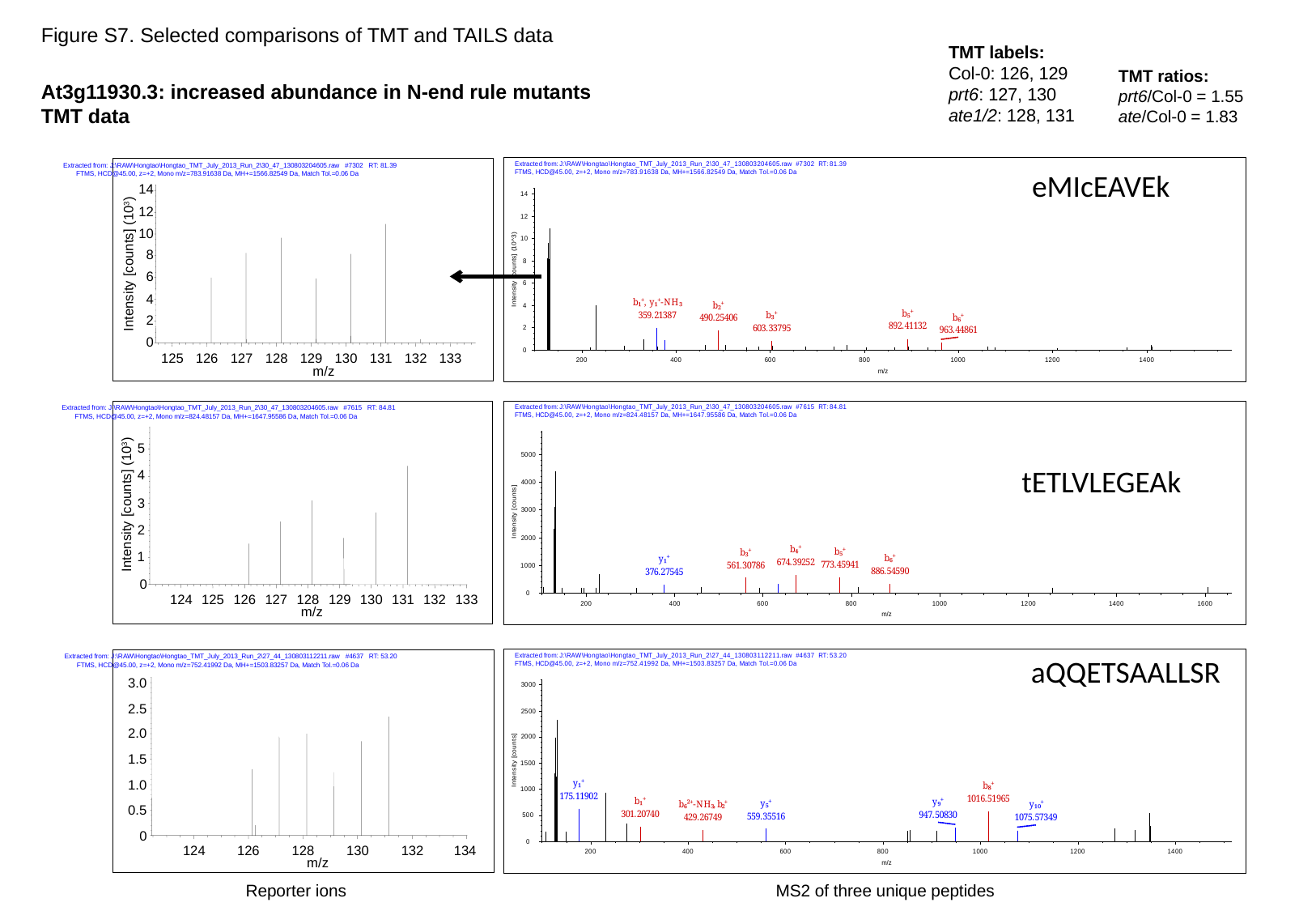

Figure S7. Selected comparisons of TMT and TAILS data
TMT labels:
Col-0: 126, 129
prt6: 127, 130
ate1/2: 128, 131
TMT ratios:
prt6/Col-0 = 1.55
ate/Col-0 = 1.83
At3g11930.3: increased abundance in N-end rule mutants
TMT data
 Extracted from: J:\RAW\Hongtao\Hongtao_TMT_July_2013_Run_2\30_47_130803204605.raw #7302 RT: 81.39
| eMIcEAVEk |
| --- |
 FTMS, HCD@45.00, z=+2, Mono m/z=783.91638 Da, MH+=1566.82549 Da, Match Tol.=0.06 Da
14
12
10
8
Intensity [counts] (103)
6
4
2
0
125
126
127
128
129
130
131
132
133
m/z
 Extracted from: J:\RAW\Hongtao\Hongtao_TMT_July_2013_Run_2\30_47_130803204605.raw #7615 RT: 84.81
 FTMS, HCD@45.00, z=+2, Mono m/z=824.48157 Da, MH+=1647.95586 Da, Match Tol.=0.06 Da
5
| tETLVLEGEAk |
| --- |
4
Intensity [counts] (103)
3
2
1
0
124
125
126
127
128
129
130
131
132
133
m/z
 Extracted from: J:\RAW\Hongtao\Hongtao_TMT_July_2013_Run_2\27_44_130803112211.raw #4637 RT: 53.20
| aQQETSAALLSR |
| --- |
 FTMS, HCD@45.00, z=+2, Mono m/z=752.41992 Da, MH+=1503.83257 Da, Match Tol.=0.06 Da
3.0
2.5
2.0
1.5
1.0
0.5
0
124
126
128
130
132
134
m/z
Reporter ions
MS2 of three unique peptides

## Slide 2
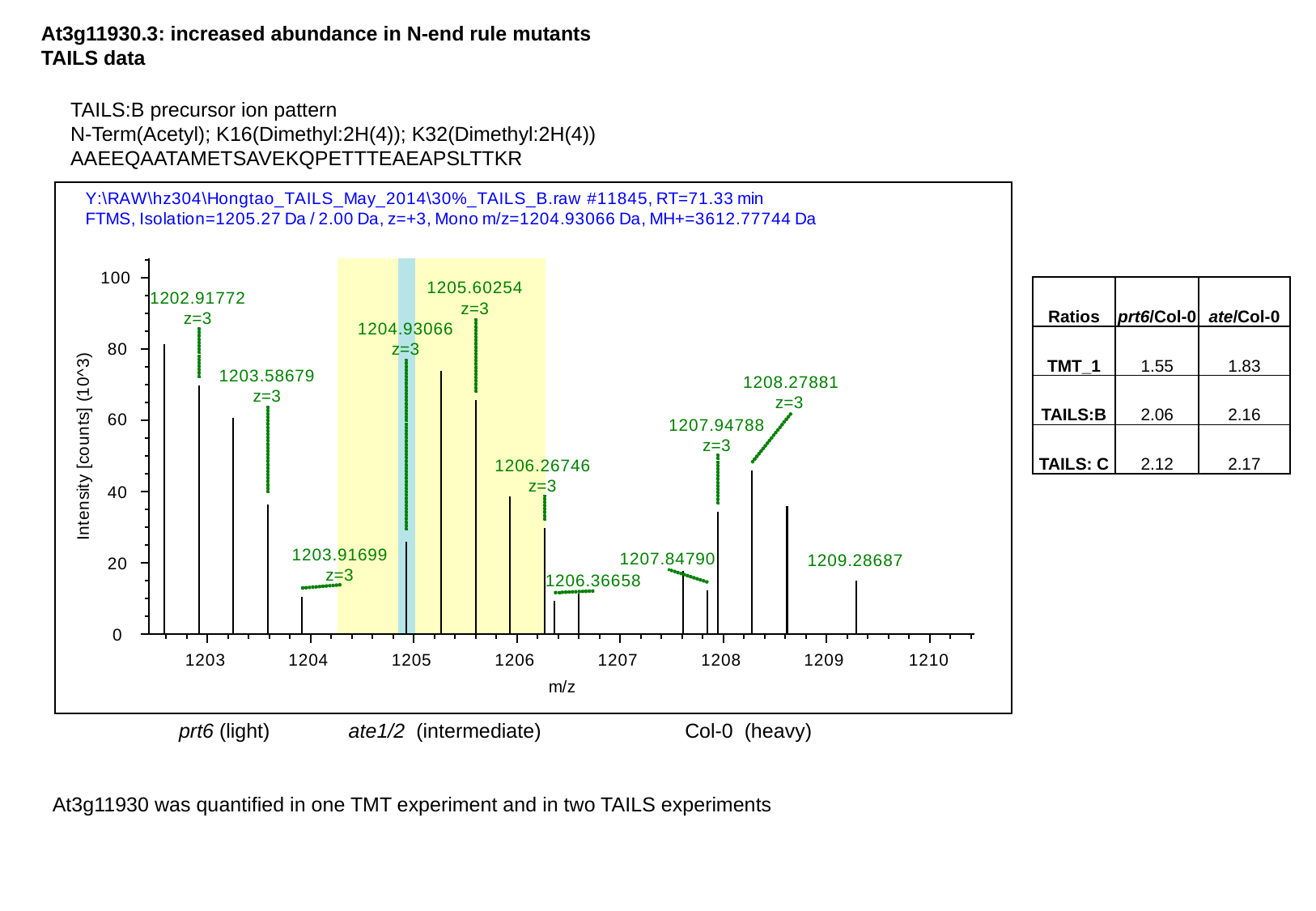

At3g11930.3: increased abundance in N-end rule mutants
TAILS data
TAILS:B precursor ion pattern
N-Term(Acetyl); K16(Dimethyl:2H(4)); K32(Dimethyl:2H(4))
AAEEQAATAMETSAVEKQPETTTEAEAPSLTTKR
| Ratios | prt6/Col-0 | ate/Col-0 |
| --- | --- | --- |
| TMT\_1 | 1.55 | 1.83 |
| TAILS:B | 2.06 | 2.16 |
| TAILS: C | 2.12 | 2.17 |
prt6 (light)
ate1/2 (intermediate)
Col-0 (heavy)
At3g11930 was quantified in one TMT experiment and in two TAILS experiments

## Slide 3
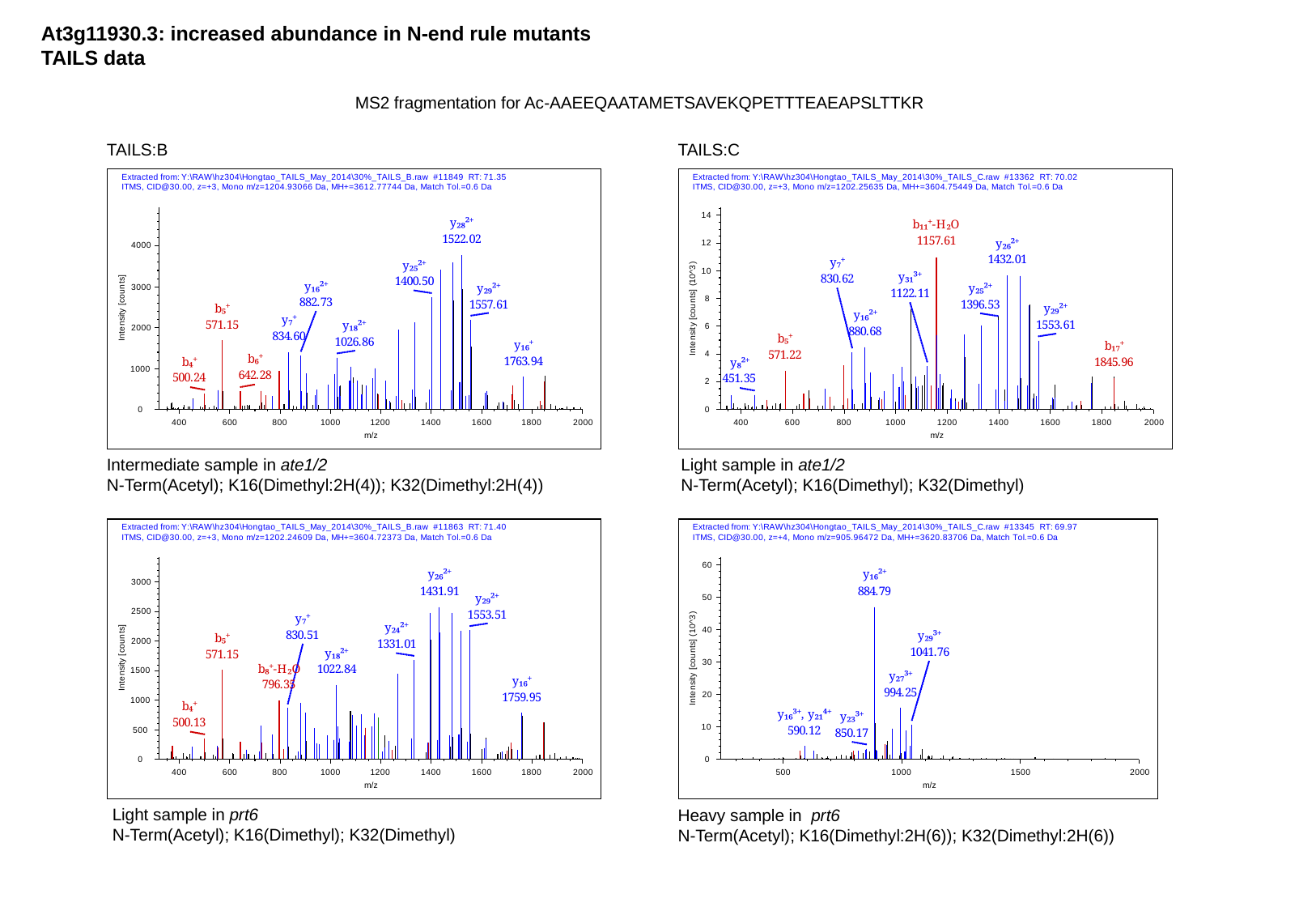

At3g11930.3: increased abundance in N-end rule mutants
TAILS data
MS2 fragmentation for Ac-AAEEQAATAMETSAVEKQPETTTEAEAPSLTTKR
TAILS:B
TAILS:C
Light sample in ate1/2
N-Term(Acetyl); K16(Dimethyl); K32(Dimethyl)
Intermediate sample in ate1/2
N-Term(Acetyl); K16(Dimethyl:2H(4)); K32(Dimethyl:2H(4))
Light sample in prt6
N-Term(Acetyl); K16(Dimethyl); K32(Dimethyl)
Heavy sample in prt6
N-Term(Acetyl); K16(Dimethyl:2H(6)); K32(Dimethyl:2H(6))

## Slide 4
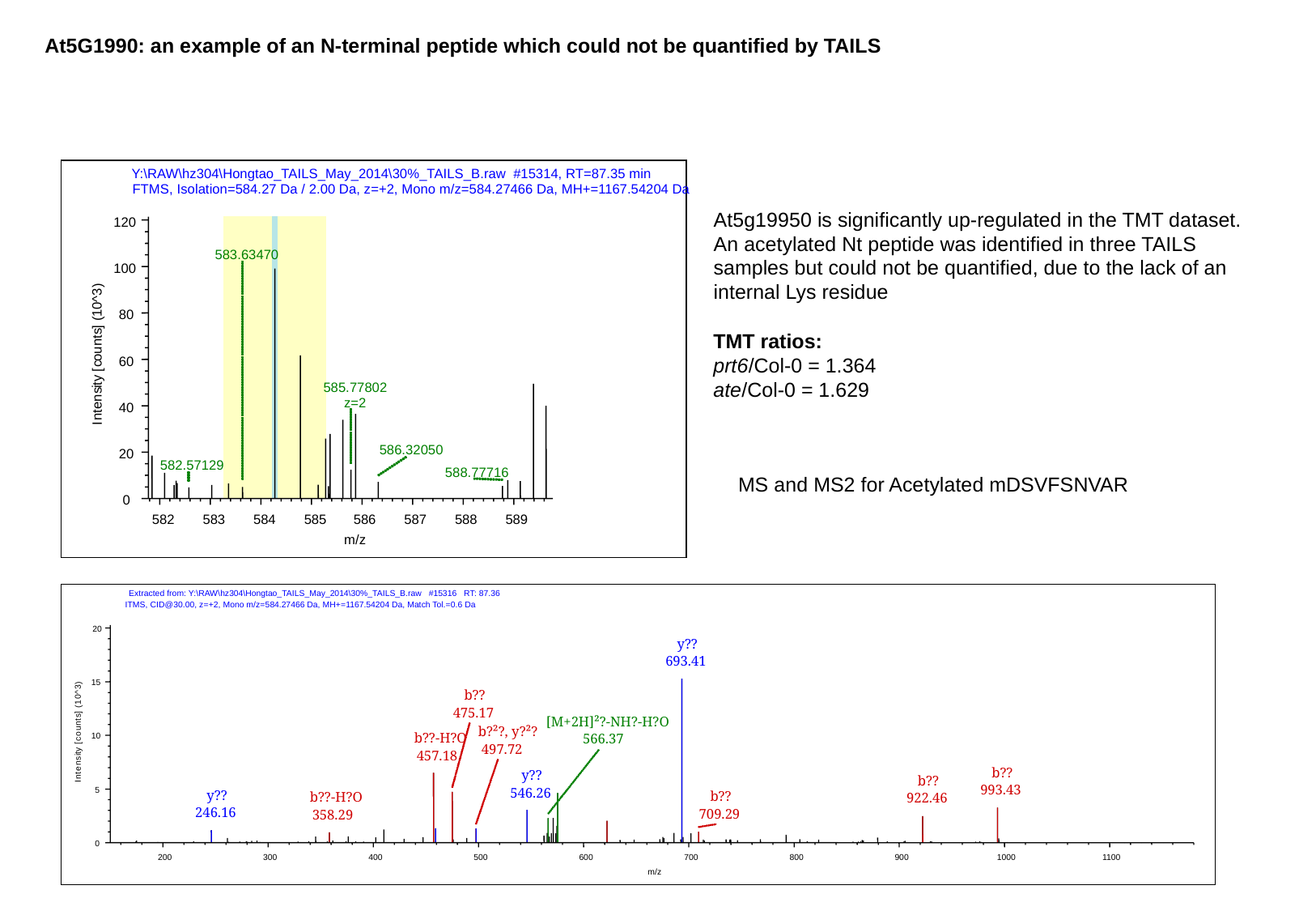

At5G1990: an example of an N-terminal peptide which could not be quantified by TAILS
 Y:\RAW\hz304\Hongtao_TAILS_May_2014\30%_TAILS_B.raw #15314, RT=87.35 min
 FTMS, Isolation=584.27 Da / 2.00 Da, z=+2, Mono m/z=584.27466 Da, MH+=1167.54204 Da
At5g19950 is significantly up-regulated in the TMT dataset. An acetylated Nt peptide was identified in three TAILS samples but could not be quantified, due to the lack of an internal Lys residue
120
583.63470
100
)
3
^
0
1
80
(
]
s
TMT ratios:
prt6/Col-0 = 1.364
ate/Col-0 = 1.629
t
n
u
o
60
c
[
y
t
i
585.77802
s
n
z=2
e
40
t
# MS and MS2 for Acetylated mDSVFSNVAR
n
I
586.32050
20
582.57129
588.77716
0
582
583
584
585
586
587
588
589
m/z
[M+2H]²?-NH?-H?O
b?²?, y?²?
566.37
497.72
b??
y??
b??
993.43
546.26
y??
b??
b??-H?O
922.46
246.16
709.29
358.29
 Extracted from: Y:\RAW\hz304\Hongtao_TAILS_May_2014\30%_TAILS_B.raw #15316 RT: 87.36
 ITMS, CID@30.00, z=+2, Mono m/z=584.27466 Da, MH+=1167.54204 Da, Match Tol.=0.6 Da
20
y??
693.41
)
15
3
^
b??
0
1
(
475.17
]
s
t
n
u
o
b??-H?O
10
c
[
y
t
457.18
i
s
n
e
t
n
I
5
0
200
300
400
500
600
700
800
900
1000
1100
m/z
