# Supplementary material for: Quantitative proteomics analysis of the Arg/N-end rule pathway of targeted degradation in Arabidopsis roots
Source: Proteomics. 2015 Apr 17;15(14):2447–57. doi: 10.1002/pmic.201400530 (PMC4692092; doi:10.1002/pmic.201400530)
Supplement: Supplementary file 1 [file pmic0015-2447-sd1.zip › pmic8103-sup-0008-text.docx]

**Supplementary Material**

**Supplementary Methods**

**Root proteome preparation and Tandem Mass Tag^TM^ (TMT) labelling**

Protein was extracted from roots of 5 d old Col-0 (wild type), *prt6-5* and *ate1/2* seedlings in buffer containing 8M urea, 50 mM triethylammonium bicarbonate (TEAB) (pH 8.5), 1x Complete Mini protease inhibitor Cocktail (Roche), 1 x PhosSTOP Phosphatase Inhibitor Cocktail (Roche) and 100 µM MG-132 (Sigma). Protein was quantified using Bradford’s reagent. Protein aliquots (250 µg) were reduced with 10 mM TCEP (Tris[2-carboxyethyl] phosphine) in 50 mM TEAB, alkylated with 17 mM iodoacetamide and diluted with 50 mM TEAB to give a final concentration of 1.5M urea. Protein was precipitated with six volumes of pre-chilled (-20°C) acetone overnight then suspended in 100 μl 100 mM TEAB and digested with 2.5 µg trypsin (T8658-1VL, from bovine pancreas; Sigma) for 4h. A further 2.5 µg trypsin was added for 16 h. Peptide aliquots (100 µg) were labelled with amine-reactive TMTsixplex^TM^ reagents (Thermo Scientific), according to the manufacturer’s protocol. Two biological replicates, each with two technical replicates were performed: in the first experiment, Col-0 was labelled with TMT^6^-126, -129, *prt6-5* with TMT^6^-127, -130 and *ate1/2* with TMT^6^-128,-131. Labels were swapped in the second experiment (Col-0 labelled with TMT^6^-127, -130; *prt6-5* with TMT^6^-128, -131 and *ate1/2* with TMT^6^-126, -129). Equal amounts of peptides were mixed, dried down and fractionated as described in Groen et al. (2014) with modifications. Half of the peptides (300 µg) were resuspended in 0.1 ml 20 mM ammonium formate (pH 10.0). Peptides were loaded onto an Acquity bridged ethyl hybrid C18 UPLC column (Waters; 2.1 mm i.d. x 150 mm, 1.7 µm particle size), and profiled with a 70 min gradient [A: 20 mM ammonium formate (pH 10.0).B: A+80% Acetonitrile, 0−10 min: 5% buffer B, 10−35 min: 5-35% buffer B, 35-60 min: 35%-70% buffer B, 60-61 min 70%-100% B, 61-67 min 100% B, 67-70min 0% B] at a flow-rate of 0.244 ml/min. Chromatographic performance was monitored by sampling eluate with a diode array detector (Acquity UPLC, Waters), scanning between wavelengths of 200 and 400 nm. Samples were collected in 1 min increments from 25 min and reduced to dryness by vacuum centrifugation. 34 fractions were collected for each LC run and the fractions were pooled together in the following way: fraction 1 with fraction 18, fraction 2 and fraction 19, fraction 3 and fraction 20, etc. This ensured the 17 pooled fractions of peptides had a good range of hydrophobicities for each LC-MS/MS run.

**LC-MS/MS for TMT experiments**

Dried fractions from the high pH RP separations were resuspended in 30 µl 0.1% formic acid and placed into a glass vial. 1 µl of each fraction was injected by the HPLC autosampler and separated by the LC. LC-MS/MS experiments were performed using a nanoAcquity UPLC (Waters Corp., Milford, MA) system and an LTQ Orbitrap Velos hybrid ion trap mass spectrometer (Thermo Scientific, Waltham, MA). Separation of peptides was performed by reverse-phase chromatography using a Waters reverse-phase nano column (BEH C18, 75 µm id x 250 mm, 1.7 µm particle size) at flow rate of 300 nl/min. Peptides were initially loaded onto a pre-column (Waters UPLC Trap Symmetry C18, 180 µm id x 20mm, 5 µm particle size) from a nanoAcquity sample manager with 0.1% formic acid for 3 minutes at a flow rate of 5 µl/min. After this period, the column valve was switched to allow the elution of peptides from the pre-column onto the analytical column. Solvent A was water + 0.1% formic acid and solvent B was acetonitrile + 0.1% formic acid. The gradient employed for the two hour run was 5-30% B in 100 minutes, followed by a washing and re-equilibration step (120 minute total run). For the three hour run, the gradient was 10% B after 10 minutes, 25% B after 120 minutes and 45% B after 155 minutes, followed by wash and equilibration steps to give a total run time of 180 minutes.

The LC eluent was sprayed into the mass spectrometer by means of a nanospray source. All *m/z* values of eluting ions were measured in the Orbitrap Velos mass analyzer, set at a resolution of 30000. Data dependent scans (Top 20) were employed to automatically isolate and generate fragment ions by higher-energy collisional dissociation (HCD, Normalised collision energy: 40, stepped by 10%) in the HCD cell, before the fragment ions were passed into the Orbitrap (7500 resolution), via the C-trap, for mass analysis, resulting in the generation of MS/MS spectra. Ions with charge states of 2+ and above were selected for fragmentation.

**Mass spectrometry data analysis for TMT experiments**

Raw data were searched against TAIR10 database (FASTA db: TAIR10_20110103_rgm.fasta, Version: 2.3, Number of sequences: 27417) using Mascot 2.4 (Matrix Science) and Proteome Discoverer™ version 1.4.1.14 (DBVersion: 79; Thermo Scientific), employing Top 10 peaks filter node and percolator nodes. As filters, high peptide confidence corresponding to an FDR < 1%, search engine rank 1 and mascot ion score 20 were used.

TMT experiments included reporter ions quantifier employing trypsin enzyme specificity with a maximum of one missed cleavage with carbamidomethylation of Cys (+ 57.0214 Da) as a fixed modification and TMT 6plex (Thermo) at Lys and N-termini, oxidized methionine (+ 42.0105 Da) as variable modifications. Mass tolerances were set to 10 ppm for MS and 0.06 Da for MS/MS. For quantification, integration window tolerance was set to 0.0075 Da. Each reporting ion was divided by the sum of total ion and normalized by medians of the samples (Table S1).

**Statistical Analysis of TMT data**

The normalised protein quantification data were analysed for each of the 2408 proteins with two biological replicates by weighted analysis of variance (ANOVA) (Sprent, 1969; Draper and Smith, 1981), which also takes account of the two technical replicates per biological replicate to ensure that tests are based on residual *biological* variation. Firstly, the ANOVA was performed with a blocking factor for the two experiments, but this was found to be significant (p < 0.05, F-test) for only 9 of the 2408, and so was omitted to provide an F-test for significance between Arabidopsis genotypes on 2 and 3 degrees of freedom (df). The weight for an observation was the square root of the number of unique peptides plus the inverse of the estimated variance for the quantification, so that more weight was given to quantification with greater associated information and lower variance. Predicted means were output based on the weights used. No transformation of the data was required. For proteins with overall significant (p < 0.1, F-test) differences between genotypes, a post-ANOVA t-test on 3 df was used to compare *prt6* and *ate1/2* to the Col-0 control. Statistically significant results were then restricted to those with >1.3 fold-change to ensure biological significance. The GenStat (16^th^ edition, © VSN International Ltd, Hemel Hempstead, UK) statistical package was used for analysis.

**LC-MS for TAILS experiments**

Samples were resuspended in 0.1% formic acid and placed into a glass vial. Each sample was injected by the HPLC autosampler and separated by the LC method detailed below. All LC-MS/MS experiments were performed using a nanoAcquity UPLC (Waters Corp., Milford, MA) system and an LTQ Orbitrap Velos hybrid ion trap mass spectrometer (Thermo Scientific, Waltham, MA). Separation of peptides was performed by reverse-phase chromatography using a Waters reverse-phase nano column (BEH C18, 75 µm id x 250 mm, 1.7 µm particle size) at flow rate of 300 nl/min. Peptides were initially loaded onto a pre-column (Waters UPLC Trap Symmetry C18, 180 µm i.d x 20mm, 5 µm particle size) from the nanoAcquity sample manager with 0.1% formic acid for 3 minutes at a flow rate of 5 µl/min. After this period, the column valve was switched to allow the elution of peptides from the pre-column onto the analytical column. Solvent A was water + 0.1% formic acid and solvent B was acetonitrile + 0.1% formic acid. The gradient employed was 5-30% B in 100 minutes, followed by a washing and re-equilibration step (120 minute total run).

The LC eluent was sprayed into the mass spectrometer by means of a New Objective nanospray source. All *m/z* values of eluting ions were measured in the Orbitrap Velos mass analyzer, set at a resolution of 30000. Data dependent scans (Top 20) were employed to automatically isolate and generate fragment ions by collision-induced dissociation in the linear ion trap, resulting in the generation of MS/MS spectra. Ions with charge states of 2+ and above were selected for fragmentation.

**Identification of arginylated proteins**

To identify post-translational arginylation of N-termini, a third search was attempted with raw files using semi-ArgC enzyme specificity with a maximum of 1 missed cleavage with carbamidomethylation of Cys as fixed modification and the following variable modifications: dimethylation light (+28.031 Da), intermediate (+32.056 Da) and heavy (+36.076Da) at Lys and Arg_dimethylation light (+184.13241 Da), intermediate (+188.15752 Da) and heavy (+192.17678) at N-termini, oxidized methionine (+ 15.994915), asparagine deamidation ( + 0.984016) and glutamine deamidation ( + 0.984016). Several N-terminal peptides were identified as putatively modified by Arg_dimethylation but none of these could be confirmed by manual check.

**References**

Draper, N.R., Smith, H., *Applied regression analysis*, Wiley, New York 1981.

Groen, A.J., Sancho-Andrés, G., Breckels, L.M., Gatto, L., Aniento, F., Lilley, K.S. Identification of Trans-Golgi Network Proteins in *Arabidopsis thaliana* Root Tissue J. Proteome Res., 2014, 13, 763–776

Sprent, P., *Models in regression and related topics*, Methuen and Co. Ltd, London 1969.

**Supplementary figures**

**Figure S1 Overview of the eukaryotic Arg/N-end rule and the Ac/N-end rule pathways**

Redrawn after Gibbs et al. (2014). The Arg/N-end rule pathway recognises non-acetylated, neo-N-termini of protein substrates. Proteins are synthesised with Nt methionine (M), but neo-N-termini can be generated by endopeptidase (EPase) cleavage. In yeast, proteins with primary destabilising residues are targeted for proteasomal degradation by the N-recognin, Ubr1p which has distinct binding sites for Type 1 residues (R,K,H; in red) and Type 2 residues (F,Y,W,L,I; in blue). In contrast, plants have at least three distinct N-recognins: PRT6 and PRT1 which recognise basic and aromatic N-termini, respectively and a third as-yet uncharacterised E3 ligase which recognises N-termini initiating with L and I. Acidic N-terminal amino acids (D,E) are considered to be secondary destabilising residues, since they can be post-translationally arginylated by the action of arginyl t-RNA transferase (ATE) enzymes. Nt amides (N,Q) are tertiary destabilising residues, since they can enter the Arg/N-end rule following enzymatic conversion to D and E, respectively by N-terminal amidases. In mammals and plants, the removal of Nt methionine (M) by methionine aminopeptidases (MetAPs) to reveal a cysteine also creates an tertiary destabilising residue because cysteine is also subject to arginylation, following enzyme-catalysed oxidation which requires the presence of NO (green box). Cysteine is stabilising in yeast which does not synthesise NO. The Ac/N-end rule pathway recognises N-terminal acetylated residues. MetAPs cleave adjacent to small amino acid residues at position 2 (A,G,V,S,T,C,) which are then susceptible to acetylation by N-terminal acetyl transferases (NATs). Nt M can also be acetylated. Acetylated proteins are generally stable in the correctly folded and oligomeric state, but misfolding or complex misassembly may reveal Nt degrons which are recognised by Ac/N-recognins, leading to degradation by the 26S proteasome. The yellow shaded areas of the Ac/N-end rule pathway and the proposed degradation of MΦ proteins by the Arg/N-end rule pathway are based on data from yeast (Kim, H.-K., Kim, R.-R., Oh, J.-H., Cho, H., Varshavsky, A., et al. The N-terminal methionine of cellular proteins as a degradation signal. *Cell* 156, 1-12).

**Figure S2. Enrichment and identification of N-terminal peptides by TAILS**

A. Numbers of unique dimethyl-labelled peptides identified before (pre-TAILS) and after enrichment by TAILS. Note that the semi ArgC/dimethylation search is not a stringent search for free Nt peptides when applied to the pre-TAILS dataset. Also, this dataset includes peptides which are not assigned to a protein group. B. Venn diagram showing overlap of putative free (dimethylated) Nt peptides identified in pre-TAILS and TAILS samples. C, D. Intensity plots showing distribution of PSMs; q values are indicated by the colour code shown to the right of the plot: all are less than 0.01 (FDR <1%). Total datasets are shown for pre-TAILS (C) and TAILS (D).

**Figure S3 Abundance of *PRT6*, *ATE1* and *ATE2* transcripts in different plant tissues and organs**

Data were obtained from public databases of Arabidopsis microarray experiments using eYFP Browser (Toufighi, K., Brady, S.M., Austin, R., Ly, E., Provart, N.J. 2005 The Botany Array Resource: e-Northerns, expression angling and promoter analyses. *Plant J*. 43, 153-163).

**Figure S4 Expression of MC-GUS in *ate1 ate2* seedlings**

Transgenic lines stably expressing *35S*::MC-GUS in the *ate1/2* background were stained for GUS activity after 5 days’ growth. A, whole seedling (scale bar = 0.5 cm); B, root (scale bar = 50 µm); C, cotyledon (scale bar = 200 µm).

**Figure S5 Frequency of different amino acid residues at position 2 in Arabidopsis predicted ORFs**

Predicted protein coding sequences were obtained from TAIR10.

**Figure S6 Analysis of free N-terminal peptides**

Occurrence of different N-terminal amino acid residues in neo-N-terminal peptides. N-terminal peptides from Col-0, *prt6* and *ate1/2* roots were enriched using TAILS. Only neo-N-terminal peptides where the N-terminus corresponds to residue ≥ 3 of the predicted translated protein were analysed. The bars represent means ± SE of the percentage occurrence of different amino acids at the new N-terminus.

**Figure S7 Selected comparisons of TMT and TAILS data**

For comparative purposes, all expression ratios from the TMT experiment are listed in Table S3 (TAILS data, “N-termini” tab, columns K and L). In Table S1, the significantly regulated proteins with over 1.3 fold change were highlighted as bold if N-terminal peptide information is also available.

**Table S1 Proteins identified and quantified using TMT labelling**

***Sheet 1: N-end rule TMT proteomics data***

Quantified protein files were exported from Proteome discoverer. Protein accession, description, score, coverage, number of proteins in the each protein group, unique peptides, total peptides and PSM identified for each protein group are listed. Values given represent two technical replicates of reporter ion intensity normalized with the median, with quantification account and variability. Further descriptions of the proteins list in the first column, including amino acid numbers of the proteins, MW molecular weight of the proteins and calculated isoelectric points of the proteins are also listed. The normalized reporter ion intensity and the unique peptides are in Bold. Two biological replicates are listed separately and technique replicate are named as sample_1 and sample_2 for biological experiment 1; sample_3 and sample_4 for experiment 2.

***Sheet 2: Statistical analysis of protein ratios***

The normalised protein quantification data for 2408 proteins with two biological replicates were analysed as described above. P values of F-test are colour-coded as indicated. Each column shows the accession of the protein groups (B), description (C), P values of F-test (D) and P values of t-test comparing *prt6* and *ate1/2* to the Col-0 control (E and F). Ratio (G and H), standard error (I and J) and log2 transformed ratio (K and L) comparing *prt6* and *ate1/2* to the Col-0 control are shown. Microarray data from Gibbs, D.J., Lee, S.C., Isa, N.M., Gramuglia, S., Fukao, T. et al. Homeostatic response of plants to hypoxia is regulated by the N-end rule pathway. *Nature* 2011 479, 415-418 are included for comparison. 1357 quantified proteins in a single experiment are listed in red (rows 2415-3771) and 23 identified proteins without quantification information are listed in purple (rows 3772-3794).

***Sheet 3: Significantly up 1.3 fold in experiments***

Proteins significantly up-regulated 1.3 fold in *prt6* and *ate1/2* relative to Col-0 are shown. Microarray data are shown for comparison. Proteins are marked in **bold** where Nt peptide information is present in the TAILS dataset (Table S3)

***Sheet 4: Significantly down 1.3 fold in experiments***

Proteins significantly down-regulated 1.3 fold in *prt6* and *ate1/2* relative to Col-0 are shown. Microarray data are shown for comparison. Proteins are marked in **bold** where Nt peptide information is present in the TAILS dataset (Table S3)

***Sheet 5 MC proteins***

Proteins with cysteine as second residue which were identified in the TMT dataset are shown.

**Table S2 Unique peptides identified in pre-TAILS and TAILS samples**

**Table S3 N-terminal peptides identified and quantified using TAILS with position information in the protein and predicted subcellular localization**

***Sheet 1: N-terminal (Nt) peptides with all quantification available***

***Sheets 2-5: Subgroups of Nt peptides***

Lists of unique acetylated Nt peptides (Ac, sheet 2) and free Nt peptides (neo, sheet 3) are derived from Sheet 1. Subcellular localisation was assigned for acetylated Nt peptides over 3 (sheet4) and for free Nt peptides over 3 (sheet 5) based on established annotation or TargetP prediction where annotation was lacking.
