# Supplementary figures and images for: Quantitative proteomics analysis of the Arg/N-end rule pathway of targeted degradation in Arabidopsis roots
Source: Proteomics. 2015 Apr 17;15(14):2447–57. doi: 10.1002/pmic.201400530 (PMC4692092; doi:10.1002/pmic.201400530)

## Slide 1
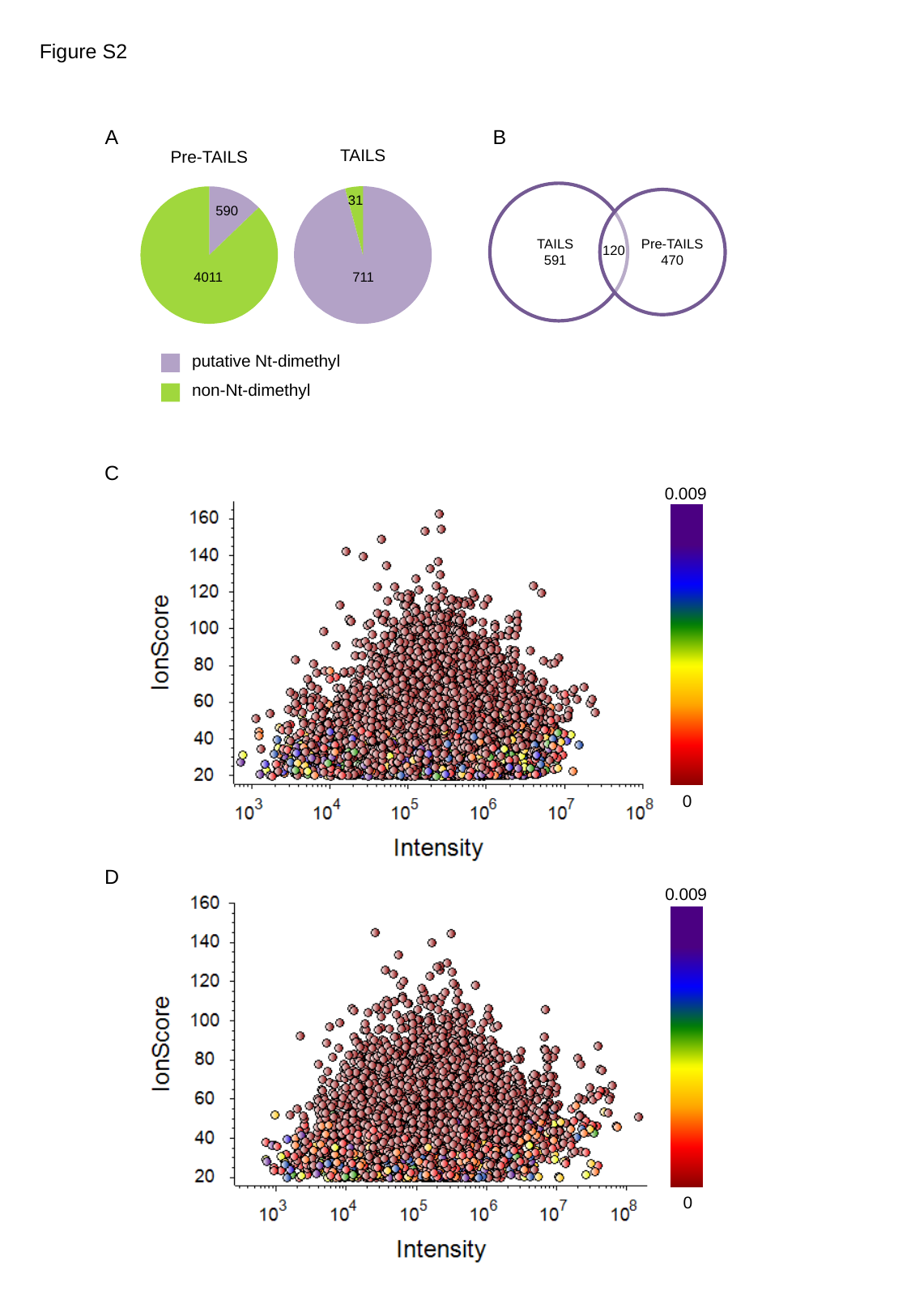

Figure S2
A
B
TAILS
Pre-TAILS
31
711
590
4011
TAILS
591
Pre-TAILS
470
120
putative Nt-dimethyl
non-Nt-dimethyl
C
0.009
0
D
0.009
0

Supplement: Supplementary file 1 [file pmic0015-2447-sd1.zip › pmic8103-sup-0002-text.pptx]

## Slide 1
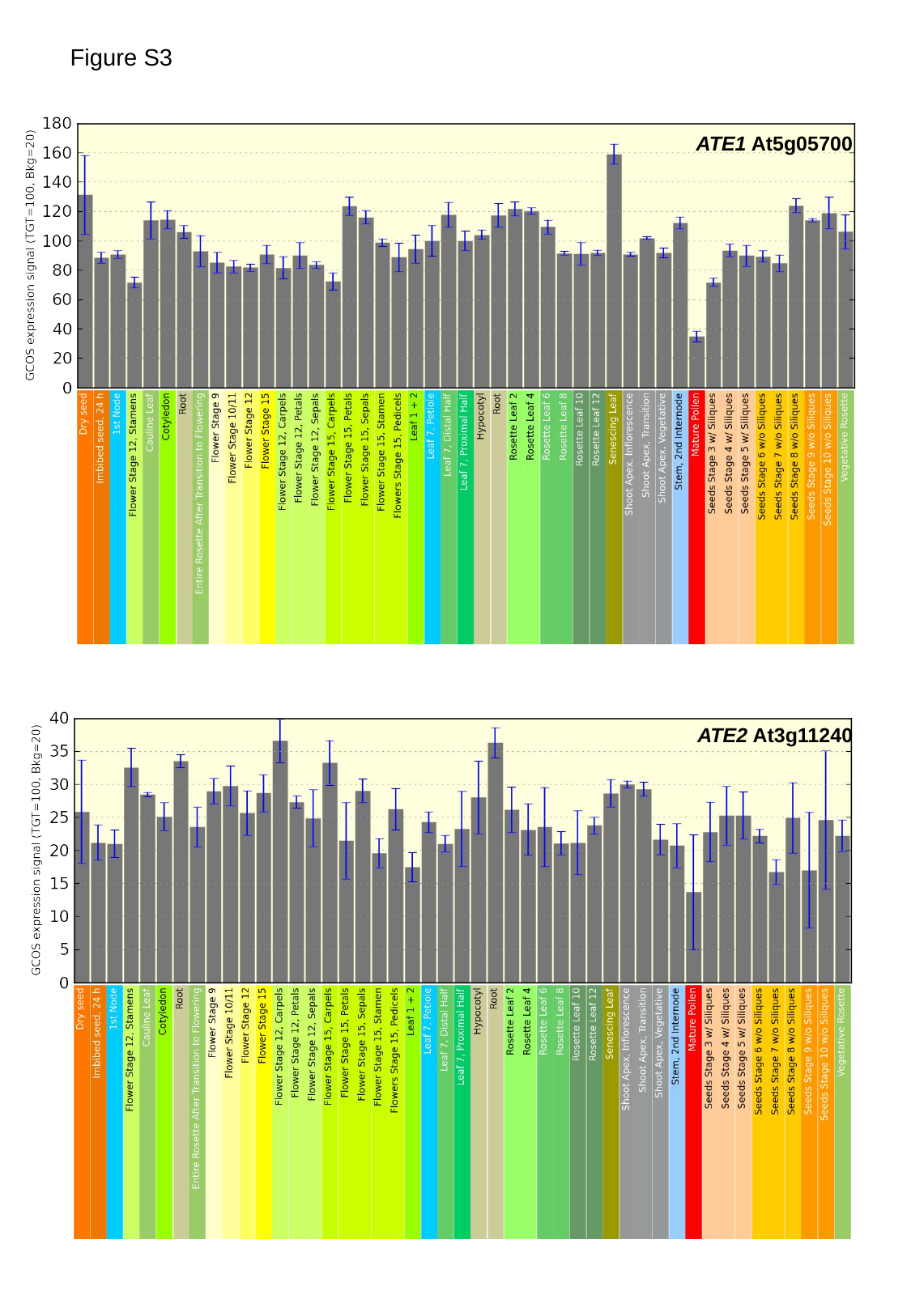

Figure S3
ATE1 At5g05700
ATE2 At3g11240

## Slide 2
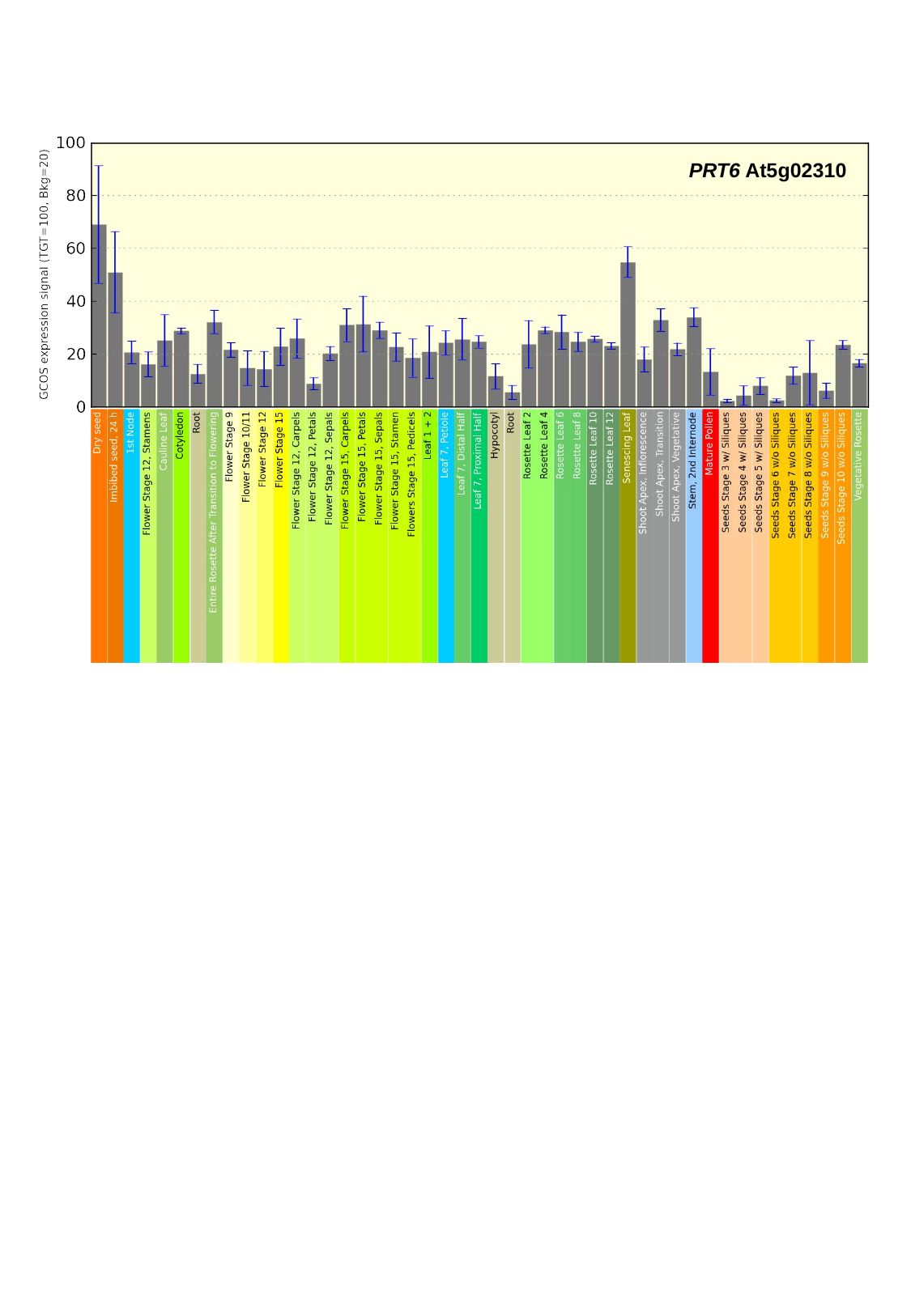

PRT6 At5g02310

Supplement: Supplementary file 1 [file pmic0015-2447-sd1.zip › pmic8103-sup-0003-text.pptx]

## Slide 1
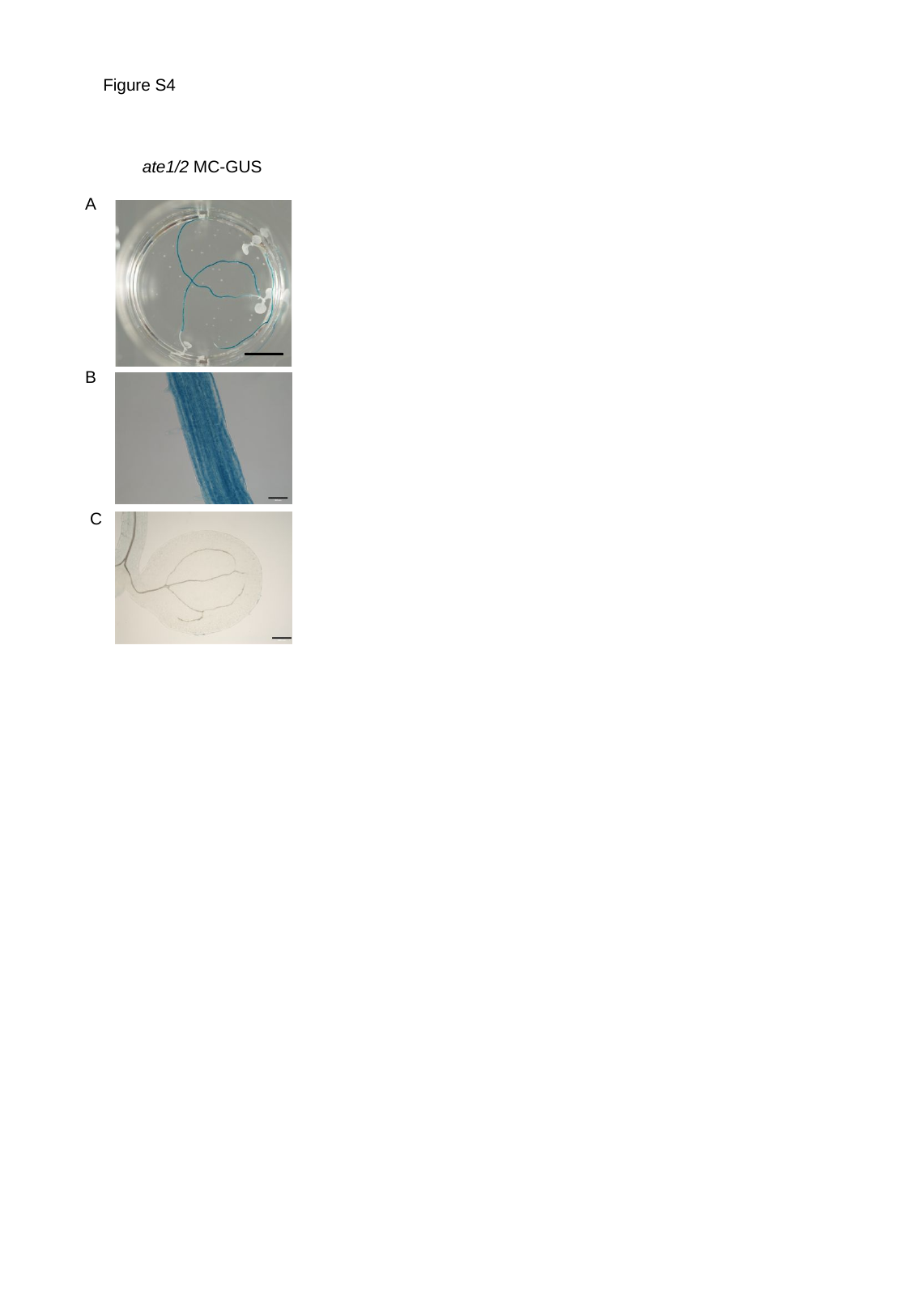

Figure S4
ate1/2 MC-GUS
A
B
C

Supplement: Supplementary file 1 [file pmic0015-2447-sd1.zip › pmic8103-sup-0004-text.pptx]

## Slide 1
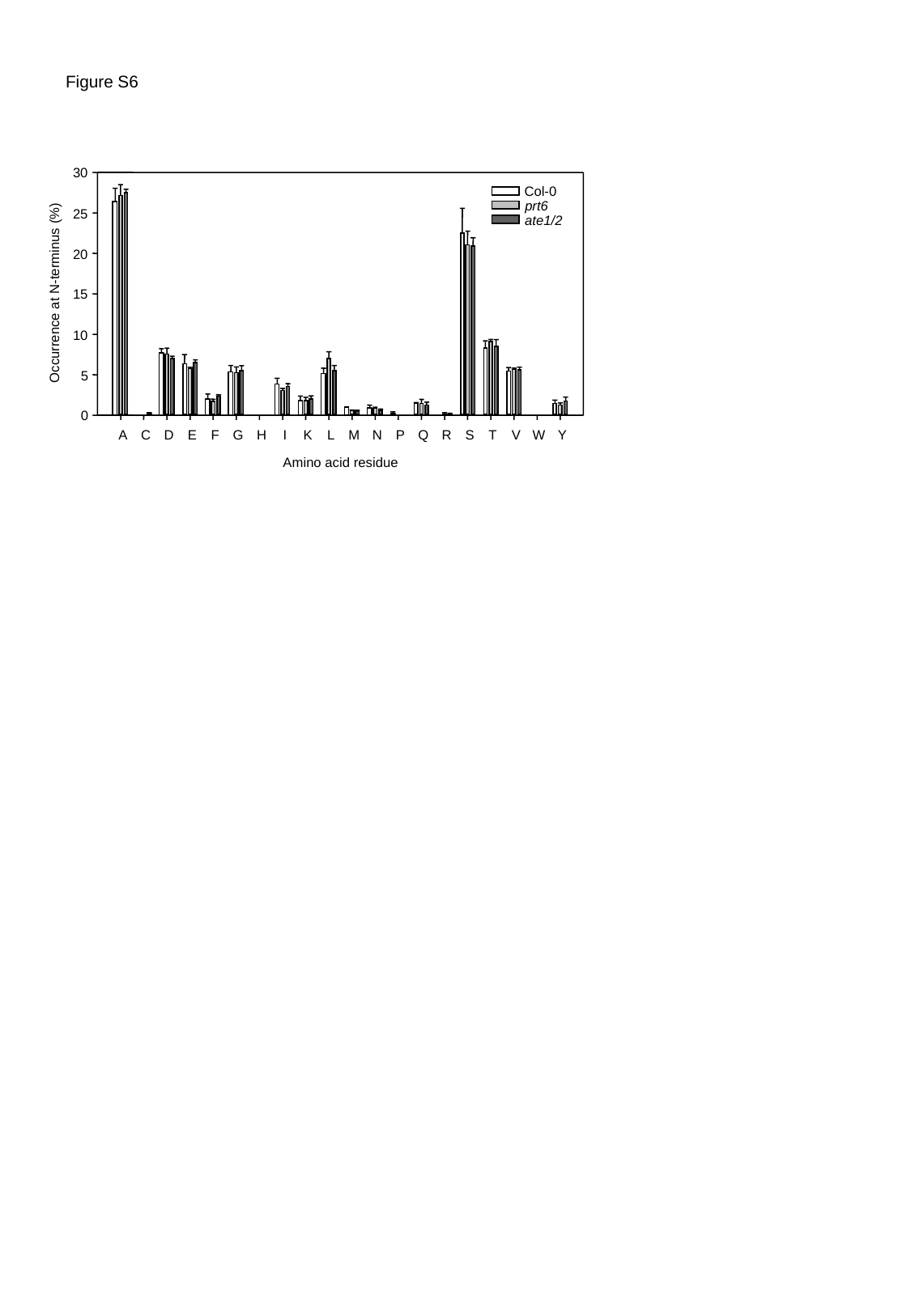

Figure S6
30
Col-0
prt6
25
ate1/2
20
Occurrence at N-terminus (%)
15
10
5
0
A
C
D
E
F
G
H
I
K
L
M
N
P
Q
R
S
T
V
W
Y
Amino acid residue

Supplement: Supplementary file 1 [file pmic0015-2447-sd1.zip › pmic8103-sup-0006-text.pptx]
